# Cell-free DNA in sepsis: from molecular insights to clinical management

**DOI:** 10.1186/s40779-025-00668-2

**Published:** 2025-12-02

**Authors:** Lei Li, Hong-Chao Huang, Yin He, Jia-Yue-Cheng Pang, Shi-Chu Xiao, Zhao-Fan Xia, Yong-Jun Zheng

**Affiliations:** 1 Department of Burn Surgery, the First Affiliated Hospital of Naval Medical University, Shanghai, 200433 China; 2 Research Unit of Key Techniques for Treatment of Burns and Combined Burns and Trauma Injury, Chinese Academy of Medical Sciences, Shanghai, 200433 China

**Keywords:** Sepsis, Cell-free DNA (cfDNA), Liquid biopsy, cfDNA scavengers, Deoxyribonuclease (DNase)

## Abstract

Sepsis is a dysregulated host response to infection that frequently results in fatal multiple organ dysfunction. Despite advances in clinical identification and management, both its incidence and mortality have remained persistently high. Emerging evidence indicates that cell-free DNA (cfDNA), as a novel biomarker and molecular therapeutic target, holds promise for improving the clinical management of sepsis. cfDNA refers to DNA fragments present in body fluids, including naked DNA, membrane-coated DNA, nucleosomes, and neutrophil extracellular traps (NETs). cfDNA is released from host cells or pathogens into body fluids through pathways, such as NETosis, mitochondrial damage, cell necrosis, apoptosis, pyroptosis, and erythroblast enucleation. The released cfDNA triggers a strong inflammatory response by activating Toll-like receptor (TLR) 9, the absent in melanoma 2 (AIM2) inflammasome, and the cyclic GMP-AMP synthase (cGAS)-stimulator of interferon genes (STING) signaling pathway. At the same time, cfDNA activates the coagulation cascade and inhibits anticoagulant and fibrinolytic systems through multiple mechanisms, resulting in microcirculatory disorders. These pathological effects are closely associated with sepsis-related organ dysfunction and poor prognosis. Elucidation of the release and pathological mechanisms of cfDNA provides a foundation for the development of targeted treatment strategies. Currently, molecular therapeutic approaches targeting cfDNA, including peptidylarginine deiminase (PAD) 4 inhibitors, pore-forming inhibitors, antioxidants, cfDNA scavengers, and deoxyribonucleases (DNases), have shown certain efficacy in treating sepsis and systemic inflammation. In terms of sepsis monitoring, compared with traditional markers, cfDNA exhibits extremely high timeliness and dynamic monitoring capability. cfDNA can simultaneously indicate the complex interplay among infection, host response, and organ damage, making it suitable for early diagnosis, prognosis assessment, treatment monitoring, organ function evaluation, and pathogen detection. Given its broad application prospects in the diagnosis and treatment of sepsis, this paper systematically elaborates on the mechanisms of cfDNA release and pathological effects in sepsis, reviews progress in cfDNA-targeted monitoring and therapeutic strategies, discusses technical challenges, and outlines potential future directions.

## Background

Sepsis is a dysregulated host response to infection that can progress to life-threatening multiple organ dysfunction syndrome (MODS) [1]. Infection induces excessive immune activation and the release of large amounts of inflammatory mediators, leading to vascular endothelial injury, capillary leakage, and coagulation dysfunction, which ultimately cause extensive organ damage and multiple complications [2]. Septic shock, disseminated intravascular coagulation (DIC), sepsis-induced cardiomyopathy, sepsis-associated encephalopathy (SAE), sepsis-associated acute lung injury (SA-ALI), sepsis-associated acute kidney injury (SA-AKI), liver dysfunction, and intestinal injury are the most common and critical manifestations of MODS caused by sepsis, and are also the principal threats faced by sepsis patients during intensive care unit (ICU) admission [3–6]. Even after surviving sepsis, patients often experience long-term complications that severely affect quality of life, including cognitive impairment, reduced physical function, and increased risk of cardiovascular events [7, 8].

To address this serious clinical challenge and reduce the global incidence and mortality of sepsis, the Surviving Sepsis Campaign (SSC), jointly initiated by the Society of Critical Care Medicine (SCCM, USA) and the European Society of Intensive Care Medicine (ESICM), has standardized and improved the diagnosis and treatment of sepsis worldwide by formulating and updating international guidelines. The latest SSC guidelines (2021) recommend de-escalation and short-course antibiotic strategies, optimization of fluid resuscitation, vasoactive drug regimens, and refined indications for glucocorticoid use, while also introducing long-term management and rehabilitation modules [9]. In 2024, SCCM introduced the “Phoenix Sepsis Score”, which evaluates 4 organ systems (respiratory, cardiovascular, coagulation, and neurological) as a new diagnostic criterion for pediatric sepsis. Compared with the previous systemic inflammatory response syndrome (SIRS)-based criterion, the Phoenix Sepsis Score enables more accurate identification of pediatric sepsis and septic shock, and demonstrates applicability across diverse healthcare resource settings [10].

Despite continuous updates to international evidence-based guidelines aimed at improving prognosis, sepsis remains one of the leading causes of morbidity and mortality worldwide [11, 12]. Globally, an estimated 48.9 million sepsis cases and 11 million related deaths occur annually [11], with mortality rates reaching 30–45% among affected patients [11, 13, 14]. An epidemiological survey across 22 countries in Asia reported a sepsis incidence of 22.4% in ICUs, with a 90-day in-hospital mortality rate as high as 36.6% [15]. These persistently high mortality rates primarily reflect the limitations of current clinical management: 1) existing monitoring systems lack precise biomarkers and dynamic assessment tools, resulting in delayed interventions; and 2) mainstream treatments, including fluid resuscitation, mechanical ventilation, and broad-spectrum antibiotics, can’t adequately target underlying pathological processes [16]. Against this background, cell-free DNA (cfDNA) has emerged as a promising biomarker and therapeutic target in sepsis, attracting considerable research and clinical interest.

cfDNA refers to DNA fragments released into body fluids by host cells or pathogens, including free DNA, membrane-bound DNA, nucleosomes, and neutrophil extracellular traps (NETs) [17–19]. According to its source, cfDNA can be classified into cell-free nuclear DNA (cf-nDNA), cell-free mitochondrial DNA (cf-mtDNA), and microbial cell-free DNA (mcfDNA) [19, 20]. Damaged and dead cells are the primary source of cfDNA, which is released through processes such as necrosis, apoptosis, pyroptosis, necroptosis, suicidal NETosis, and chromosomal instability [21–23]. In addition, cfDNA released by healthy cells during physiological processes such as erythroblast enucleation, vital NETosis, and exosome secretion also contributes to the cfDNA pool [21, 23]. Multiple studies have demonstrated that cfDNA levels are elevated in critical conditions such as sepsis, acute respiratory distress syndrome, trauma, and ischemia–reperfusion injury, and are positively correlated with inflammatory responses [24, 25]. In these settings, cfDNA functions not only as a byproduct and biomarker of inflammation and tissue damage but also as a pathological mediator that exacerbates inflammatory cascades and tissue injury [24, 25]. As a result, cfDNA has attracted considerable interest in the context of diagnosing and managing critical illnesses.

Recognized as a classical damage-associated molecular pattern (DAMP), cfDNA provokes strong inflammatory responses through the activation of specific pattern recognition receptors (PRRs) [25]. These receptors, located on the surface or within immune cells, detect conserved microbial motifs [pathogen-associated molecular patterns (PAMPs)] as well as endogenous danger signals, DAMPs, thereby initiating innate immune responses [25]. In sepsis, Toll-like receptor (TLR) 9, absent in melanoma 2 (AIM2), and cyclic GMP-AMP synthase (cGAS) are the principal PRRs responsible for sensing cfDNA [26]. Furthermore, cfDNA promotes thrombosis and microcirculatory dysfunction in sepsis by activating coagulation pathways while inhibiting anticoagulant and fibrinolytic systems [27, 28]. This cfDNA-driven interplay between inflammation and coagulation establishes a self-amplifying vicious cycle, leading to SIRS, DIC, and extensive organ damage (Table 1) [22, 27, 29–52]. Over recent decades, therapeutic research focusing on inflammation and coagulation regulation in sepsis has led to the development of single-target drugs, such as PRR inhibitors, cytokine antagonists, tissue factor pathway inhibitor (TFPI), and antithrombin [53–55]. The tissue factor (TF) pathway, also known as the extrinsic coagulation pathway, represents one of the initiating steps in coagulation. This pathway is triggered when TF binds to activated coagulation factor VII (FVIIa) to form a complex, which subsequently activates factor X (FX) and factor IX (FIX), thereby initiating the coagulation cascade [56]. Although some drugs demonstrated modest efficacy in early trials, most failed to reduce mortality in phase II/III clinical trials significantly. A critical reason lies in the complexity of sepsis, which involves numerous molecular networks and signaling pathways, rendering single-target interventions insufficient [53–55]. Consequently, cfDNA, as a pivotal pathological mediator bridging inflammation and coagulation in sepsis, is considered to possess unique therapeutic potential.

**Table 1:** Pathological effects of cfDNA in sepsis

Pathological effects	Pathological mechanisms	cfDNA types	Models/Cells/Samples	Pathogens/Stimuli	Interventions on pathways	Main findings	References
Inflammatory activation	Activation of the TLR9-MyD88 pathway	mtDNA	Mice; NR8383 cells	LPS; mtDNA	TLR9 siRNA	mtDNA promotes the release of inflammatory factors by activating the TLR9-MyD88-NF-κB pathway, exacerbating LPS-induced SA-ALI	[30]
		mtDNA	Mice	LPS; mtDNA	TLR9 inhibitor ODN2088	mtDNA is released in a TLR4-dependent manner during LPS stimulation, subsequently contributing to SA-ALI and systemic inflammation via the TLR9-MyD88-p38 MAPK pathway	[31]
		mtDNA	Mice	LPS; CLP; Exogenous mitochondrial debris	*TLR9* knockout; DNases	mtDNA is massively released after CLP and drives systemic inflammation, splenocyte apoptosis, and renal tubular injury via the TLR9 pathway	[32]
		mtDNA; CpG ODNs; Bacterial DNA; Plasmodium DNA	Mice; Human, mouse, and chimpanzee RBCs; Mouse splenic macrophages	Cecal slurry model; Legionella; Toxoplasma gondii; mtDNA; CpG ODNs; Bacterial DNA; Plasmodium DNA	*TLR9* knockout; TLR9 antibody	cfDNA promotes innate immune activation in sepsis by activating TLR9 on the surface of RBCs	[33]
		CpG ODNs	Mice; Mouse BMDCs; Mouse splenic γδT cells	CLP; CpG ODNs	*TLR9* knockout; *IL-17A* knockout	CpG ODNs activate TLR9 in dendritic cells, mediating IL-17A production by γδT cells and thereby promoting SA-AKI	[34]
		Bacterial DNA	Mice; Mouse BMDMs	LPS; CLP; Dextran sulfate solution; Bacterial DNA	Endosomal acidification inhibitor chloroquine/monensin	In septic mice, serum bacterial DNA is partly derived from gut translocation. Bacterial DNA enhances LPS-induced inflammatory responses by activating the TLR9 signaling pathway, predominantly mediated by macrophages	[35]
	Activation of the AIM2 inflammasome pathway	mtDNA	Mice; Mouse cardiomyocytes	LPS	shAIM2 plasmid	LPS triggers AIM2 inflammasome activation by inducing mPTP opening and mtDNA efflux in cardiomyocytes, thereby driving inflammatory responses and cardiomyocyte death	[36]
		NET-DNA	Mice; Mouse lung neutrophils	CLP	DNase I; PAD4 inhibitor Cl-amidine	CD177⁺ neutrophils predominate in septic lungs and exacerbate SA-ALI by releasing excessive NETs, which subsequently activate the AIM2 inflammasome	[37]
	Activation of the cGAS-STING pathway	dsDNA	Mice; Mouse RAW264.7 macrophages	LPS; Purified cfDNA; Synthetic dsDNA analog poly (dA:dT)	Cationic nanoparticles	cfDNA exacerbates the inflammatory response in SA-ALI by activating the cGAS-STING pathway in macrophages	[38]
		mtDNA	Mice; Mouse macrophages	LPS; mtDNA; Mitochondrial oxidative phosphorylation uncoupling agent CCCP	*cGAS* knockout; *STING* knockout; STING agonist; NLRP3 overexpression	LPS stimulation elevates cytosolic mtDNA levels in macrophages. This mtDNA promotes macrophage pyroptosis and inflammatory responses by activating the cGAS-STING-NLRP3 pathway, thereby driving SA-ALI	[39]
		mtDNA	Mice; HK-2 cells	LPS	cGAS inhibitor RU.521; STING agonist DMXAA; Ethidium bromide	mtDNA contributes to the pathogenesis of SA-AKI by activating the cGAS-STING signaling pathway in renal tubular epithelial cells, which subsequently promotes NLRP3 inflammasome activation	[40]
		mtDNA	Mice; Mouse Kupffer cells; Mouse hepatocytes	LPS	*STING* knockout; STING agonist DMXAA; DRP1 shRNA; DNase I	In Kupffer cells, LPS induces mitochondrial fission by mediating DRP1 phosphorylation, thereby promoting mtDNA release. The liberated mtDNA activates the STING signaling pathway, ultimately exacerbating inflammatory responses and sepsis-associated liver injury	[41]
		mtDNA	Mice; Mouse BMDMs; Mouse small intestinal lamina propria dendritic cells	CLP; mtDNA; Cyclic dinucleotides	*STING* knockout; STING agonist DMXAA; DNase I	In sepsis, mtDNA exacerbates inflammatory responses, intestinal epithelial cell apoptosis, and intestinal barrier dysfunction by activating the STING pathway in lamina propria dendritic cells, culminating in bacterial translocation, systemic inflammation, and MODS	[42]
		mtDNA	Mice; Mouse microglia	CLP	*STING* knockout; STING inhibitor C176; Idebenone; Dimethyl fumarate	mtDNA mediates microglial pyroptosis and amplifies neuroinflammation by activating the cGAS-STING-NLRP3 inflammasome pathway, thereby propelling the progression of SAE	[43]
		mtDNA	Mice; Human pulmonary microvascular endothelial cells	LPS; CLP; mtDNA; cGAMP	*cGAS* knockout; *GSDMD* knockout	LPS induces mitochondrial membrane pore formation and mtDNA release by activating GSDMD. The released mtDNA inhibits endothelial cell proliferation and promotes inflammatory injury by activating the cGAS-STING pathway	[44]
		mtDNA; Bacterial DNA	Mice; Mouse BMDMs	LPS; CLP; Bacterial DNA	*cGAS* knockout	mtDNA and bacterial DNA induce additional pro-inflammatory effects in LPS-activated macrophages by activating the cGAS signaling pathway	[45]
Thrombus formation	Activation of the intrinsic coagulation pathway	NET-DNA	Human neutrophils; Healthy human plasma; Septic patient plasma	LPS; PMA	DNase I; PAD4 inhibitor Cl-amidine; Corn trypsin inhibitor	cfDNA enhances thrombin generation by activating the intrinsic coagulation pathway and is closely associated with the abnormal activation of the coagulation system during sepsis	[46]
		NET-DNA; Human neutrophil DNA	Human neutrophils; Human plasma	IL-8; Glucose oxidase	DNase I	NETs and cfDNA recruit HK and FXII to activate the contact system, subsequently triggering the intrinsic coagulation pathway and inducing the release of the inflammatory mediator bradykinin	[47]
	Mediation of the procoagulant phenotype transition	dsDNA; ssDNA	HUVECs	dsDNA; ssDNA	TLR9 inhibitor E6446	Low-molecular-weight dsDNA and ssDNA activate HUVECs via the TLR9 pathway, triggering vWF release and upregulating TF expression	[22]
		–	Mice; Mouse neutrophils; Mouse platelets	CLP; cGAMP	*STING* knockout; STING inhibitor H151	The cGAS-STING pathway exacerbates thrombosis and NETosis in sepsis by promoting platelet activation and granule secretion	[48]
	Modulation of clot structure and impairment of fibrinolysis	Genomic DNA	Healthy and septic human plasma	Genomic DNA	DNase I	cfDNA impairs fibrinolysis by either directly binding to plasmin or forming an inactive ternary complex through simultaneous interaction with fibrin and plasmin, thereby hindering normal fibrin degradation by plasmin	[27]
		Human neutrophil DNA	Purified protein system	Human neutrophil DNA	DNase I	cfDNA and histones enhance the mechanical stability and shear resistance of clots by structurally modifying fibrin networks, while simultaneously inhibiting plasmin activity through binding to fibrin degradation products, ultimately establishing a fibrinolysis-resistant state	[49]
		dsDNA; ssDNA; ODNs	Purified protein system	cfDNA	DNase I	High concentrations of cfDNA reduce the efficiency of fibrin dissolution by stabilizing fibrin architecture and competing with plasmin for fibrin binding sites	[50]
	Impairment of the anticoagulant system	Nucleosome DNA	Mice; Human neutrophils, human platelets and THP-1 cells; Mouse neutrophils and mouse platelets	Nucleosomes; *Escherichia coli*	DNase I	DNA within nucleosomes promotes the co-localization of NE with TFPI via its anionic surface, thereby enhancing NE-mediated proteolytic degradation of TFPI. This attenuates TFPI’s inhibitory effects on both the TF-FVIIa complex and the prothrombinase complex	[51]
		Histone-complexed DNA	Human plasma	–	–	Elevated levels of histone-complexed DNA demonstrate a significant association with increased circulating syndecan-1 (indicating endothelial glycocalyx damage), abnormal platelet aggregation, and coagulopathy	[52]

*MyD88* myeloid differentiation primary response 88, *NF-κB* nuclear factor kappa-B, *mtDNA* mitochondrial DNA, *LPS* lipopolysaccharide, *siRNA* small interfering RNA, *MAPK* mitogen-activated protein kinase, *DNase* deoxyribonuclease, *CpG* cytidine-phosphate-guanosine, *ODNs* oligodeoxynucleotides, *BMDCs* bone marrow-derived dendritic cells, *BMDMs* bone marrow-derived macrophages, *mPTP* mitochondrial permeability transition pore, *NET-DNA* neutrophil extracellular trap-derived DNA, *PAD* peptidylarginine deiminase, *dsDNA* double-stranded DNA, *ssDNA* single-stranded DNA, *STING* stimulator of interferon genes, *NLRP3* NOD-like receptor protein 3, *DMXAA* 5,6-dimethylxanthenone-4-acetic acid, *DRP1* dynamin-related protein 1, *shRNA* short hairpin RNA, *CLP* cecal ligation and puncture, *cGAMP* cyclic GMP-AMP, *GSDMD* gasdermin D, *PMA* phorbol 12-myristate 13-acetate, *IL* interleukin, *HK* high-molecular-weight kininogen, *FXII* factor XII, *HUVECs* human umbilical vein endothelial cells, *vWF* von Willebrand factor, *NE* neutrophil elastase, *cfDNA* cell-free DNA, *cGAS* cyclic GMP-AMP synthase, *SA-ALI* sepsis-associated acute lung injury, *CCCP* carbonyl cyanide m-chlorophenylhydrazone, *MODS* multiple organ dysfunction syndrome, *SAE* sepsis-associated encephalopathy, *NETs* neutrophil extracellular traps, *TLR* Toll-like receptor, *TF* tissue factor, *TFPI* tissue factor pathway inhibitor, *FVIIa* factor VIIa, *AIM2* absent in melanoma 2, *CD177⁺* CD177 antigen positive, *THP-1 cells* human acute monocytic leukemia cell line, *RBCs* red blood cells, *SA-AKI* sepsis-associated acute kidney injury, *shAIM2* plasmid short hairpin AIM2 plasmid

In addition, the field of critical care medicine has been actively seeking biomarkers for rapid and accurate early diagnosis, risk stratification, prognosis assessment, and treatment response monitoring. As a novel biomarker, cfDNA is gaining increasing attention in critical illness monitoring due to its direct correlation with cellular damage [24]. Multiple studies have shown that plasma cfDNA levels in septic patients rise significantly early in the disease course (averaging 41.2-fold higher than in healthy controls) and exhibit a strong positive correlation with organ dysfunction and mortality risk [57–60]. Consequently, cfDNA is regarded as a sensitive biomarker for early diagnosis and prognosis assessment in sepsis. A prospective study published in 2024 conducted long-term dynamic monitoring of plasma cfDNA levels in septic patients. The results revealed that cfDNA levels were markedly elevated during the acute phase, gradually decreased with clinical improvement, and returned to or fell below healthy control levels at 6 and 12 months [59]. Furthermore, several studies have demonstrated a positive correlation between plasma cfDNA levels and disease severity in septic patients [20, 60, 61]. These findings suggest that dynamic monitoring of plasma cfDNA levels may serve as a valuable tool for tracking disease progression, enabling clinicians to evaluate treatment efficacy and promptly adjust therapeutic strategies. Moreover, cfDNA-based epigenetic profiling allows accurate tracing of tissue-of-origin, providing a powerful approach for organ-specific functional monitoring [62]. Meanwhile, metagenomic next-generation sequencing (mNGS) of mcfDNA has revolutionized diagnostics, enabling the establishment of a rapid, broad-spectrum, and high-precision pathogen detection platform [63]. These advancements in cfDNA-based diagnostic technologies provide critical guidance for localizing injured organs, identifying pathogen types, and selecting precise antibiotic therapies in sepsis. Therefore, plasma cfDNA demonstrates multiple clinical utilities in sepsis, encompassing early warning, prognosis assessment, and therapeutic guidance.

This review systematically integrates the pathological mechanisms of cfDNA in sepsis and its targeted intervention strategies, with particular emphasis on cfDNA release mechanisms and clinical advances, aiming to provide novel insights for the development of a precision diagnostic and treatment paradigm for sepsis.

## cfDNA in sepsis pathology

### Mechanisms of cfDNA release

In healthy individuals, host cfDNA accounts for 90–95% of the total cfDNA, whereas mcfDNA is present only in trace amounts [64]. Although mcfDNA levels rise markedly during sepsis, host cfDNA still constitutes the predominant fraction [58, 65]. Host cfDNA originates from multiple tissues, including leukocytes, hepatocytes, and intestinal epithelial cells, with neutrophils being the most important contributors [66]. Specifically, plasma levels of leukocyte-derived cfDNA (predominantly from neutrophils) in septic patients are more than 20-fold higher than in healthy controls [66].

From a mechanistic perspective, the formation and degradation of NETs constitute the primary source of cfDNA in sepsis [22, 67]. During sepsis, systemic hyperinflammation strongly activates neutrophils, leading to extensive NET formation and deposition in organs such as the lungs [68], liver [69], kidneys [70], brain [71], and intestines [72]. In addition, cfDNA is also released via cell death pathways, including apoptosis, necrosis, necroptosis, and pyroptosis, as well as through physiological processes in healthy cells, such as erythroblast enucleation and exosome secretion [21, 23].

#### NETosis

NETs are web-like DNA structures released by neutrophils, enriched with histones, granular proteins, and cytosolic proteins [73]. NETs exert a dual role in innate immunity. On the one hand, they capture pathogens and expose them to high concentrations of antimicrobial components, thereby effectively eliminating bacteria, fungi, viruses, and parasites [74–77]. cfDNA, as the structural backbone of NETs, facilitates the entrapment of pathogens via its mesh-like architecture, restricting dissemination, and promoting clearance [78]. This suggests that cfDNA may exert beneficial effects during the early anti-infective phase of sepsis. On the other hand, excessive NET formation can override this protective function, driving systemic inflammation and immunothrombosis [79, 80]. In sepsis, aberrant neutrophil activation results in massive NETs release, causing severe tissue injury and ultimately MODS [81].

Neutrophil extracellular trap-derived DNA (NET-DNA), a distinct form of cfDNA, is the key mediator of the pro-inflammatory and procoagulant effects [82]. NET-DNA is usually derived from nuclear DNA (nDNA) [83], although under certain conditions it may contain mtDNA [84]. In septic patients, cfDNA is predominantly low-molecular-weight cfDNA (LMW-cfDNA), with NET-DNA as its primary source. Plasma cfDNA fragments, including both host cfDNA and mcfDNA, mainly range between 150 and 200 bp [85–87]. Patients with severe infections and poor outcomes typically exhibit shorter cfDNA fragments, characterized by a predominance of LMW-cfDNA [86, 88]. Compared with high-molecular-weight cfDNA (HMW-cfDNA), LMW-cfDNA displays significantly stronger pro-inflammatory and pro-thrombotic activity, which likely explains its strong association with poor sepsis outcomes [22, 89]. For example, intracellular nuclear factor kappa-B (NF-κB) pathway activation is markedly higher in LMW-cfDNA-treated group than in HMW-cfDNA-treated group, indicating the weaker pro-inflammatory activity of HMW-cfDNA [89]. Furthermore, unlike LMW-cfDNA, HMW-cfDNA and intact NETs neither accelerate fibrin generation nor induce endothelial TF expression or von Willebrand factor (vWF) release, suggesting limited pro-thrombotic potential [22, 90, 91]. Currently, no universally accepted definition exists to distinguish LMW-cfDNA from HMW-cfDNA. For instance, Ngo et al. [22] defined HMW-cfDNA as fragments > 50 kb, while LMW-cfDNA ranged between 100 and 500 bp. Raghuram et al. [89] also classified HMW-cfDNA as > 50 kb but defined LMW-cfDNA within 300–3000 bp. Saelee et al. [92] considered LMW-cfDNA to typically measure 160–180 bp, whereas HMW-cfDNA generally exceeded 330 bp. In contrast, the origins of LMW-cfDNA and HMW-cfDNA are comparatively clearer. LMW-cfDNA typically arises from apoptosis, necrosis, and NET degradation, while HMW-cfDNA is mainly derived from undegraded genomic DNA released by ruptured blood cells or intact NETs structures [22, 89, 92]. Collectively, current evidence indicates that LMW-cfDNA plays a dominant role in sepsis pathology, whereas HMW-cfDNA exerts limited direct pathological activity. Nevertheless, the roles of HMW-cfDNA should not be underestimated. Established functions include serving as a reservoir for LMW-cfDNA and providing intrinsic biological information useful for organ-specific injury assessment and pathogen characterization [60, 85]. Its potential roles in inflammatory responses, coagulation regulation, and immune modulation remain insufficiently studied and warrant further investigation.

**Fig. 1:**
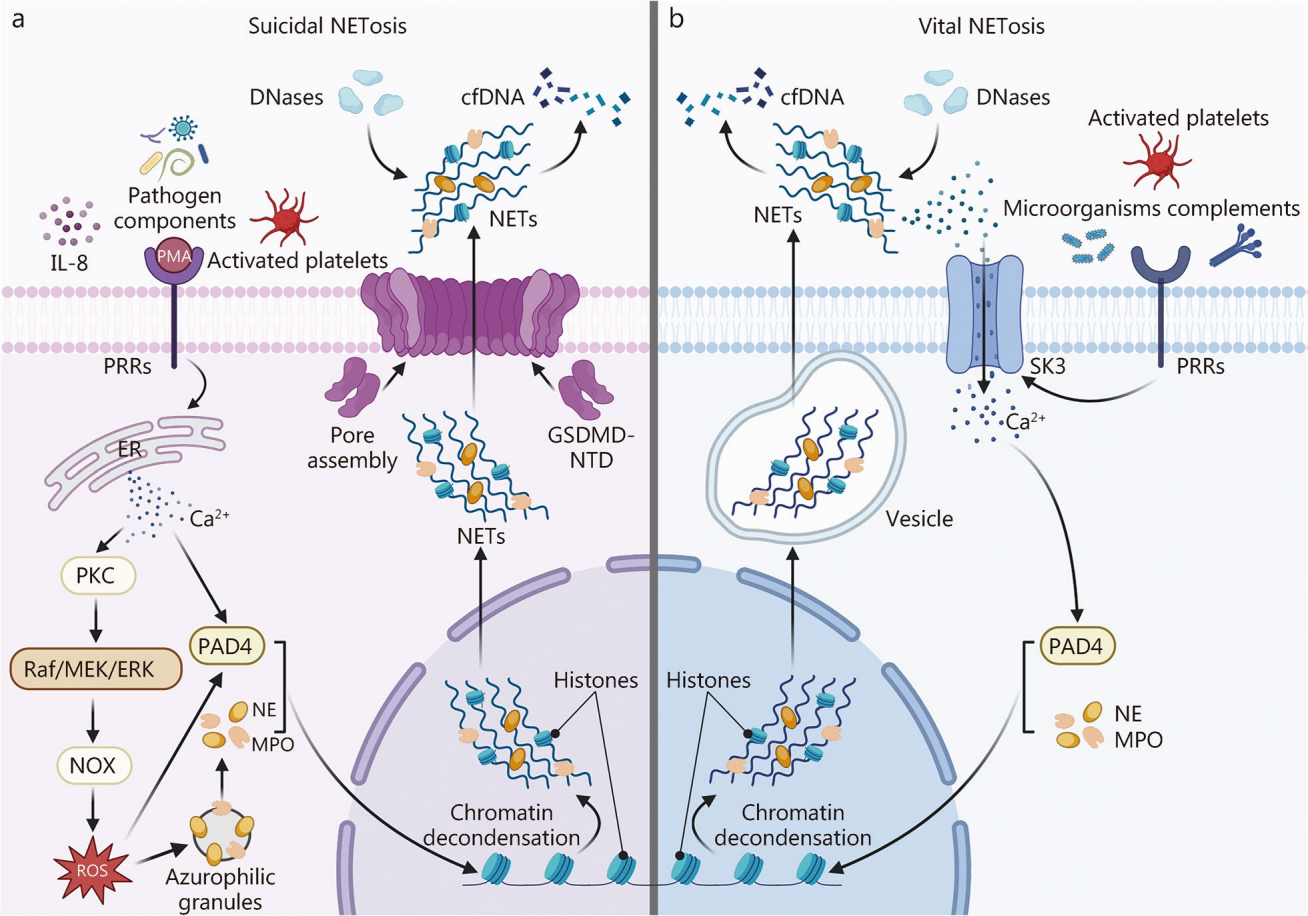
NETosis-mediated release of cfDNA. **a** Suicidal NETosis. Phorbol 12-myristate 13-acetate (PMA), interleukin (IL)-8, activated platelets, and diverse microbial components bind pattern recognition receptors (PRRs), inducing an elevation of cytosolic Ca^2+^. The Ca^2^⁺ surge activates protein kinase C (PKC), which, through the rapidly accelerated fibrosarcoma (Raf)/mitogen-activated protein kinase kinase (MEK)/extracellular signal-regulated kinase (ERK) cascade, drives the assembly and activation of nicotinamide adenine dinucleotide phosphate hydrogen oxidase (NOX). Active NOX produces large amounts of reactive oxygen species (ROS). ROS promotes the dissociation of neutrophil elastase (NE) and myeloperoxidase (MPO) from azurophilic granules and, together with Ca^2^⁺, activate peptidylarginine deiminase (PAD) 4. Activated NE, MPO, and PAD4 subsequently translocate to the nucleus, where NE degrades histones and PAD4 catalyzes histone citrullination, leading to chromatin decondensation. The decondensed chromatin mixes with granular proteins to form neutrophil extracellular traps (NETs), which are then expelled through pores formed by the N-terminal domain (NTD) of gasdermin D (GSDMD) in the plasma membrane. After NETs release, neutrophils lose membrane integrity and undergo cell death. Extracellular NETs are degraded by deoxyribonucleases (DNases) into low-molecular-weight cfDNA (LMW-cfDNA). **b** Vital NETosis. Activated platelets, complement proteins, and microbial components engage PRRs and open small-conductance calcium-activated potassium channel 3 (SK3), allowing a rapid influx of extracellular Ca^2^⁺. PAD4 is then activated and, together with NE and MPO, drives chromatin decondensation. The decondensed chromatin assembles with NE and MPO into NETs, which are rapidly extruded via vesicular budding. Throughout this process, the plasma membrane remains largely intact, allowing neutrophils to survive and retain chemotactic, phagocytic, and other defensive functions. Extracellular NETs are likewise degraded by DNases into LMW-cfDNA. ER endoplasmic reticulum

As the primary source of LMW-cfDNA in sepsis, the process of NET release is termed NETosis. Based on mechanistic differences, NETosis can be further categorized into suicidal NETosis and vital NETosis [22, 23] (Fig. 1).

Suicidal NETosis is a novel cell-death program that is distinct from necrosis and apoptosis and was first observed in neutrophils stimulated with phorbol 12-myristate 13-acetate (PMA) [93]. Various stimuli, including PMA, interleukin (IL)-8, activated platelets, and pathogen components, activate neutrophils through PRRs [94]. This activation leads to elevated intracellular Ca^2+^ levels and subsequently initiates the protein kinase C (PKC) and rapidly accelerated fibrosarcoma (Raf)/mitogen-activated protein kinase kinase (MEK)/extracellular signal-regulated kinase (ERK) signaling cascade [95, 96]. This signaling pathway promotes the assembly of the nicotinamide adenine dinucleotide phosphate hydrogen oxidase (NOX) complex, thereby triggering a respiratory burst and the robust production of reactive oxygen species (ROS) [96]. As a key regulator of suicidal NET formation, ROS coordinates several downstream processes. First, ROS facilitates the release of neutrophil elastase (NE) and myeloperoxidase (MPO) from azurophilic granules and promotes their translocation into the nucleus [97]. Within the nucleus, NE promotes chromatin decondensation by cleaving histones (e.g., histone 4 and histone 2B), and MPO synergistically enhances this process [98, 99]. Second, ROS activates PAD4 in cooperation with Ca^2+^ [100]. Activated PAD4 catalyzes histone citrullination, weakening the electrostatic interactions between histones and DNA, thereby promoting chromatin decondensation [101]. PAD4 also facilitates the disassembly of the nuclear lamin meshwork and contributes to the rupture of the nuclear envelope [101]. In parallel, ROS-induced oxidative damage to guanine bases activates DNA repair pathways involving apurinic/apyrimidinic endodeoxyribonuclease 1 and poly(ADP-ribose) polymerase 1, further promoting chromatin decondensation [102]. Upon nuclear envelope breakdown, the decondensed chromatin translocates into the cytoplasm, where it associates with granular and cytosolic proteins to form NETs. Ultimately, gasdermin D (GSDMD)-mediated pore formation in the plasma membrane and subsequent neutrophil lysis lead to the release of NETs into the extracellular space, marking the terminal step of irreversible and programmed neutrophil death [103].

Vital NETosis is a process in which neutrophils rapidly release NETs via vesicular budding while remaining viable [104]. Unlike suicidal NETosis, vital NETosis occurs independently of NOX and ROS and does not involve plasma membrane disruption or neutrophil cell death [104]. Principal stimuli for vital NETosis include activated platelets, microorganisms, and complements [23]. Following activation, neutrophils experience a Ca^2+^ influx mediated by the small-conductance calcium-activated potassium channel 3 (SK3), which subsequently activates PAD4 and induces chromatin decondensation [105, 106]. The nuclear envelope then undergoes morphological remodeling, forming vesicles that encapsulate the decondensed chromatin, which buds off from the cell to rapidly release NETs [104, 107]. Vital NETosis can also involve the extrusion of mtDNA, although the underlying mechanisms regulating its release remain incompletely understood [84].

#### Mitochondrial damage and host cell death

Mitochondria function not only as central regulators of cellular metabolism but also as pivotal mediators of various programmed cell death pathways [108]. In the context of sepsis, mitochondrial damage initiates multiple forms of cell death, including apoptosis, necrosis, necroptosis, ferroptosis, and pyroptosis [109]. Emerging evidence suggests that mitochondrial oxidative stress, impaired mitochondrial quality control (MQC), and mitochondrial membrane permeabilization (MMP) represent the 3 primary mechanisms underlying mitochondrial dysfunction and consequent cell death during sepsis [110].

During sepsis, the activities of mitochondrial NOXs (e.g., NOX4) and inducible nitric oxide synthase (iNOS) are markedly elevated, resulting in excessive generation of mitochondrial ROS (mtROS) and mitochondrial reactive nitrogen species (mtRNS) [111, 112]. In contrast, the antioxidant defense system is compromised, as evidenced by significantly decreased activities of antioxidant enzymes (e.g., superoxide dismutase and catalase) and reduced levels of antioxidant molecules (e.g., uric acid) [113, 114]. When mtROS/mtRNS production exceeds the scavenging capacity of the antioxidant system, mitochondrial oxidative stress ensues. Under these conditions, mtROS/mtRNS inflict widespread damage on mtDNA, proteins, and lipids, thereby establishing a vicious positive feedback loop [115, 116]. Mitochondrial oxidative stress compromises the integrity of mtDNA through at least 3 key pathways. First, mtROS/mtRNS induce oxidative and nitrosative modifications of nucleotide bases and the DNA backbone, resulting in base alterations and strand breaks [115, 117]. Second, mtROS reduces the expression of mitochondrial transcription factor A (TFAM) and facilitates its degradation via Lon protease, thereby disrupting the processes of mtDNA replication and transcription [118]. Third, oxidative stress impairs the proofreading function of DNA polymerase γ, leading to a higher mutation burden and decreased replication fidelity [119]. Since mtDNA encodes 13 components of the electron transport chain (ETC), along with 22 transfer RNAs and 2 ribosomal RNAs, its damage has direct consequences for mitochondrial biogenesis and cellular energy production [120]. Moreover, mtROS/mtRNS directly injures respiratory chain complexes, thereby inhibiting oxidative phosphorylation [121–123]. Under physiological conditions, these complexes are the primary intracellular source of ROS, but they generate only small amounts of ROS [124]. When damaged, however, they impair electron transport and redox balance, triggering excessive ROS production that further exacerbates mitochondrial oxidative stress [116, 124]. In addition, lipid peroxidation targets polyunsaturated fatty acids within mitochondrial membranes, compromising both membrane integrity and mitochondrial membrane potential [125]. Severe oxidative damage to mitochondria ultimately leads to MMP, activates mitochondria-dependent cell death pathways, and facilitates the release of cfDNA [108]. Before the onset of MMP, MQC serves as a crucial defensive mechanism to preserve mitochondrial stability and function.

MQC safeguards mitochondrial integrity by orchestrating a network of interconnected processes, namely mitochondrial biogenesis, mitophagy, and mitochondrial dynamics (fission and fusion), to adapt to varying cellular demands imposed by energy fluctuation, oxidative stress, or pathogen exposure [126]. Mitochondrial biogenesis supports the formation of new mitochondria and enhances their performance in response to metabolic shifts [127]. This process is primarily regulated by peroxisome proliferator-activated receptor γ coactivator-1α (PGC-1α), whose activity is finely tuned by energy-sensing signaling pathways, including AMP-activated protein kinase (AMPK), sirtuins, and calcium/calmodulin-dependent protein kinase [128]. Upon activation, PGC-1α upregulates the expression of nuclear respiratory factor (NRF)-1 and NRF-2 and directly enhances the transcriptional activity of NRF-1 [129]. These transcription factors are pivotal in promoting the expression of mitochondrial TFAM, which supports mtDNA replication and transcription, while also coordinating the synthesis of nuclear-encoded mitochondrial proteins to avoid imbalance in nucleo-mitochondrial gene expression [130]. Furthermore, PGC-1α strongly induces oestrogen-related receptor α, thereby enhancing the transcription of genes involved in the tricarboxylic acid cycle, fatty acid oxidation, and NRF-2-mediated mitochondrial pathways [131]. Mitophagy is a selective autophagic pathway that removes damaged mitochondria, whose accumulation would otherwise impair cellular function or trigger cell death [132]. The PTEN-induced putative kinase 1 (PINK1)-Parkin pathway represents the classical route of mitophagy. When mitochondrial membrane potential is lost, PINK1 accumulates on the outer mitochondrial membrane (OMM) and undergoes autophosphorylation, which enables the recruitment of the E3 ubiquitin ligase Parkin. Parkin then ubiquitinates OMM proteins, generating polyubiquitin chains that serve as molecular tags for degradation [133, 134]. These tags are recognized by mitochondrial autophagy receptors such as optineurin and nuclear dot protein 52, which facilitate the removal of damaged mitochondria via the autophagosome-lysosome system [133, 134]. In addition to this well-characterized pathway, several PINK1-Parkin-independent mechanisms have been identified, involving mediators such as FUN14 domain-containing 1, anti-apoptotic B-cell lymphoma-2 (Bcl-2) interacting protein 3, and activating molecules in autophagy and beclin 1 regulator 1. These alternative pathways highlight the mechanistic diversity of mitophagy in response to distinct cellular stressors, including oxidative stress, hypoxia, and nutrient limitation [135]. Mitochondrial dynamics regulate mitochondrial morphology, quantity, and distribution through continuous cycles of fission and fusion to adapt to metabolic changes and cellular stress [136]. Mitochondrial fission, primarily regulated by dynamin-related protein 1 (DRP1), divides impaired mitochondria into smaller and depolarized fragments that are more amenable to elimination through mitophagy [137]. In contrast, mitochondrial fusion involves the stepwise integration of the OMM and inner mitochondrial membrane (IMM), coordinated by mitofusin (MFN) proteins (1/2) and optic atrophy 1 (OPA1), respectively [138]. This fusion process facilitates the exchange of mtDNA, lipids, and metabolites between mitochondria, thereby promoting the repair of damaged organelles, improving energy efficiency, and limiting excessive mitophagy and apoptosis [138]. In the context of sepsis, these mitochondrial regulatory pathways become disrupted to varying extents. Early in the septic response, mitochondrial biogenesis is aberrantly stimulated, resulting in an overproduction of structurally compromised mitochondria that fail to meet energy demands and may instead intensify oxidative stress by increasing ROS generation [139, 140]. Simultaneously, the PINK1-Parkin-mediated mitophagy pathway is activated to remove dysfunctional mitochondria and help preserve cellular integrity and survival [141, 142]. As sepsis progresses, oxidative stress-induced TFAM depletion suppresses mitochondrial biogenesis [118]. In the late-stage sepsis, mitophagy is significantly impaired, resulting in the pathological accumulation of dysfunctional mitochondria [143, 144]. This disruption is closely associated with the cleavage of Parkin, mediated by caspase-1 activation downstream of NOD-like receptor protein 3 (NLRP3) inflammasome signaling [143, 144]. Additionally, expression of TBC1 domain family member 15 is markedly reduced in both septic patients and murine models, leading to prolonged mitochondria-lysosome interactions and further impairing mitophagic efficiency [145]. At the same time, the equilibrium between mitochondrial fusion and fission becomes increasingly skewed toward excessive fission. As a consequence, mitochondria across various tissues shift from an interconnected network to highly fragmented and punctate structures [110]. In sepsis, oxidative stress, inflammatory activation, metabolic dysregulation, and Ca^2+^ homeostasis disturbances drive this imbalance in mitochondrial dynamics, upregulating DRP1 expression and activity, while downregulating MFN1/2 and OPA1 [110, 146, 147]. The combined impact of heightened mitochondrial fission and deficient mitophagy results in the progressive buildup of damaged mitochondria, thereby exacerbating oxidative stress and promoting cell death [139].

**Fig. 2:**
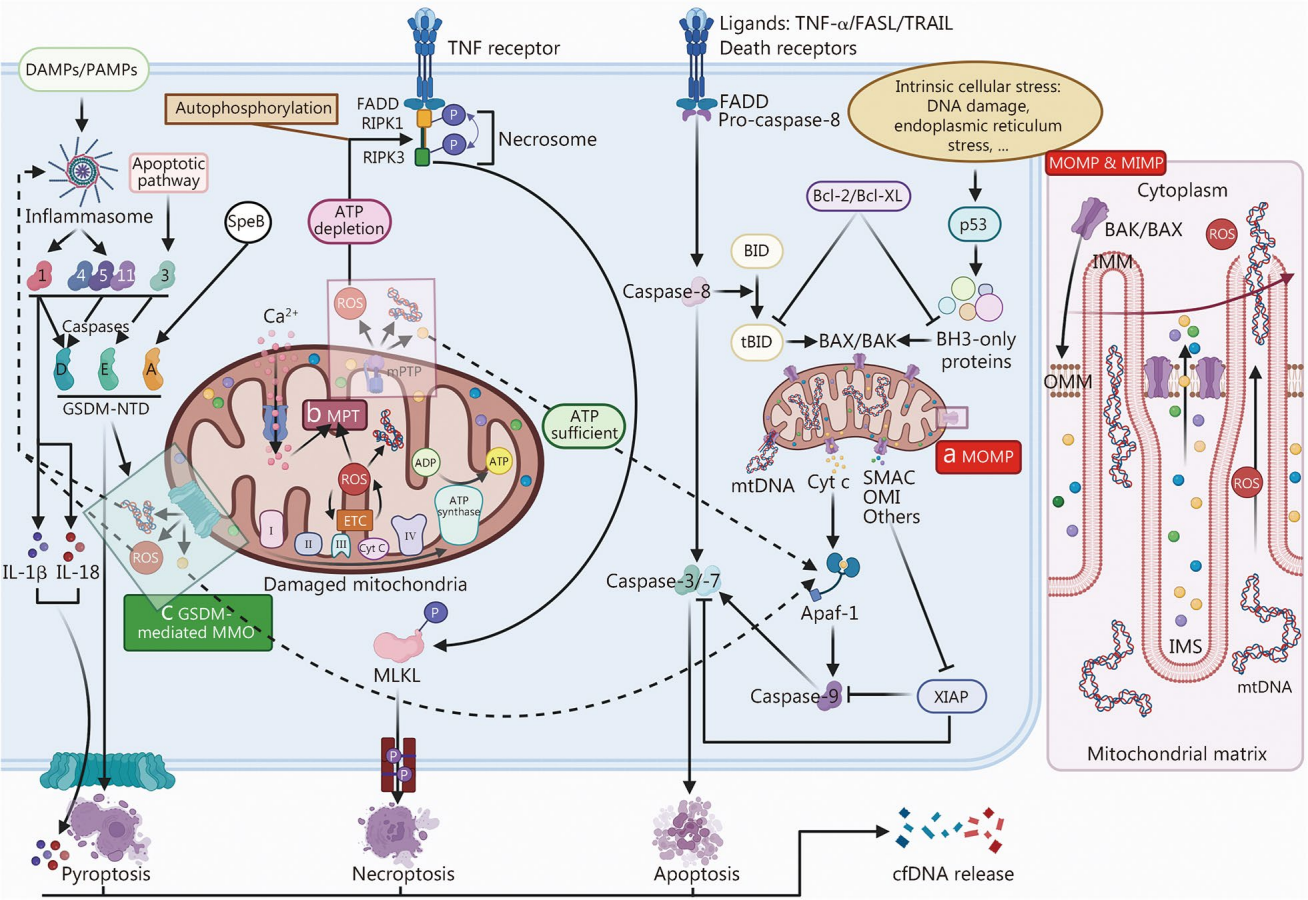
Mitochondria-associated death-mediated cfDNA release. **a** Mitochondrial outer membrane permeabilization (MOMP). In the extrinsic pathway, tumor necrosis factor (TNF)-α, Fas ligand (FASL), or TNF-related apoptosis-inducing ligand (TRAIL) engages their respective death receptors, causing Fas-associated death domain protein (FADD) to oligomerize and activate caspase-8. Active caspase-8 cleaves BH3-interacting domain death agonist (BID) to truncated BID (tBID), which drives conformational activation and oligomerization of Bcl-2-associated X protein (BAX)/Bcl-2 antagonist or killer (BAK). In the intrinsic pathway, cellular stress upregulates multiple BH3-only proteins via p53, similarly activating BAX/BAK. B-cell lymphoma 2 (Bcl-2) and B-cell lymphoma-extra large (Bcl-XL) counteract this process by sequestering BH3-only proteins. Activated BAX/BAK are embedded in the outer mitochondrial membrane (OMM) and oligomerize to produce large pores, inducing MOMP and intermembrane space (IMS) protein release. Following MOMP, BAX/BAK pores permit herniation of the inner mitochondrial membrane (IMM) into the cytosol. The exposed IMM undergoes mitochondrial inner membrane permeabilization (MIMP), releasing mitochondrial DNA (mtDNA) and mitochondrial reactive oxygen species (mtROS). Cytosolic cytochrome c (Cyt c) complexes with apoptotic protease activating factor-1 (Apaf-1) to form the apoptosome, which recruits and activates caspase-9, thereby triggering caspase-3/-7-mediated apoptosis and cell-free DNA (cfDNA) release. Second mitochondria-derived activator of caspases (SMAC) and high-temperature requirement serine peptidase A2 (OMI) amplify this cascade by neutralizing X-linked inhibitor of apoptosis protein (XIAP). **b** Mitochondrial permeability transition (MPT). Under severe stress, such as mitochondrial oxidative damage or Ca^2+^ overload, the mitochondrial permeability transition pore (mPTP) remains open, inducing MPT that expels mitochondrial contents. When adenosine triphosphate (ATP) is scarce, mtROS released during MPT drives autophosphorylation of receptor-interacting serine/threonine protein kinase (RIPK) 1, which then complexes with RIPK3 to form the necrosome. Mixed-lineage kinase domain-like protein (MLKL) is phosphorylated by the necrosome and forms pores in the plasma membrane, discharging damage-associated molecular patterns (DAMPs) like cfDNA and initiating necroptosis. When ATP is sufficient, MPT instead favors Cyt c-mediated apoptosis. **c** Gasdermin (GSDM)-mediated mitochondrial membrane opening (MMO). Inflammasomes and the apoptotic pathway activate corresponding caspases to cleave and expose the N-terminal domain of gasdermin (GSDM-NTD). Specifically, streptococcal pyrogenic exotoxin B (SpeB) can directly cleave GSDMA. The exposed GSDM-NTD oligomerises into pores, rapidly disrupting mitochondrial integrity and allowing mtDNA and Cyt c to leak into the cytosol. Cytosolic mtDNA further promotes inflammasome assembly. In addition, caspase-1 processes interleukin (IL)-1β and IL-18 to their mature forms. GSDM-NTD subsequently perforates the plasma membrane, releasing cfDNA together with cytokines and culminating in pyroptosis. Notably, GSDMA/E drive either pyroptosis or apoptosis depending on context. PAMPs pathogen-associated molecular patterns, ETC electron transport chain

When damaged mitochondria escape timely clearance by the MQC system, MMP is triggered, marking an irreversible commitment to cell death. Three principal mechanisms underlie MMP: mitochondrial outer membrane permeabilization (MOMP), mitochondrial permeability transition (MPT), and GSDM-mediated mitochondrial membrane opening (MMO) (Fig. 2). MOMP serves as the pivotal event in the intrinsic apoptotic pathway and is initiated in response to cellular stress or activation of death receptors [148]. BH3-only proteins such as BH3-interacting domain death agonist (BID), Bcl-2-interacting mediator of cell death (BIM), and p53-upregulated modulator of apoptosis (PUMA), sense these apoptotic cues and activate Bcl-2-associated X protein (BAX) and Bcl-2 antagonist or killer (BAK) through either direct engagement or inhibition of anti-apoptotic Bcl-2 family members [149]. For instance, external signals from tumor necrosis factor (TNF)-α, Fas ligand (FASL), or TNF-related apoptosis-inducing ligand (TRAIL) bind to their respective death receptors, resulting in oligomerization of Fas-associated death domain protein (FADD) and subsequent activation of caspase-8. Active caspase-8 cleaves BID into its truncated form (tBID), which then directly activates BAX and BAK via its BH3 domain [150]. Alternatively, pro-apoptotic proteins such as Bad indirectly activate BAX/BAK by neutralizing the inhibitory effects of Bcl-2 and B-cell lymphoma-extra large (Bcl-XL) [151]. Once activated, BAX and BAK translocate to the OMM, where they oligomerise to form pores that are composed of proteins or lipids [152]. This permeabilization facilitates the release of intermembrane space (IMS) proteins, including cytochrome c (Cyt c), second mitochondria-derived activator of caspases (SMAC), and high-temperature requirement serine peptidase A2 into the cytosol, initiating the caspase cascade and downstream apoptotic events [152]. Following MOMP, the mitochondrial network undergoes rapid fragmentation, and large BAX/BAK pores become evident in the OMM. These openings allow the IMM to herniate into the cytosol [153]. The herniated IMM becomes highly permeable, resulting in the leakage of mitochondrial matrix contents, including mtDNA, a process referred to as mitochondrial inner membrane permeabilization (MIMP) [154]. MPT is a pathophysiological state driven by the opening of the mitochondrial permeability transition pore (mPTP), predominantly elicited by Ca^2+^ overload, oxidative stress, elevated phosphate, and adenine nucleotide depletion [155]. Under these conditions, the IMM becomes excessively permeable, leading to mitochondrial dysfunction, collapse of cellular energy metabolism, and ultimately cell death [155]. mtDNA can escape into the cytosol through mPTP- and voltage-dependent anion channel (VDAC)-dependent routes, where it activates the NLRP3 inflammasome and the cGAS-stimulator of interferon genes (STING) pathway [156]. A fraction of mtDNA is also released extracellularly and taken up by neighboring cells via clathrin-mediated endocytosis, further amplifying the inflammatory response [156]. The mode of cell death triggered by MPT is largely determined by the intracellular availability of adenosine triphosphate (ATP) [157]. Under conditions of ATP depletion, mtROS produced during MPT initiate the autophosphorylation of receptor-interacting serine/threonine protein kinase (RIPK) 1, which subsequently forms a complex with RIPK3 to generate the necrosome [157, 158]. This complex phosphorylates mixed-lineage kinase domain-like protein (MLKL), leading to its insertion into the plasma membrane and formation of pores that facilitate the release of DAMPs, such as cfDNA, thereby promoting necroptosis [158]. In contrast, when ATP levels are adequate, MPT favors the activation of Cyt c-dependent apoptotic pathways through caspase signaling [157]. This distinction reflects the bioenergetic requirement of apoptosis: sufficient ATP supports the energy-dependent execution of programmed cell death, whereas its deficiency shifts the cellular fate toward necroptosis driven by metabolic collapse [159]. GSDM-mediated MMO represents a novel pathway for MMP. GSDMs area family of pore-forming proteins whose N-terminal domain (NTD) perforates the plasma membrane to execute pyroptosis [160]. GSDM family proteins can be activated by inflammasomes, caspases of the canonical apoptotic pathway, or streptococcal pyrogenic exotoxin B [161, 162]. Recent studies reveal that several GSDM family members rapidly translocate to mitochondrial membranes upon activation, where they form functional pores prior to plasma membrane permeabilization [163–165]. For example, the GSDMD-NTD disrupts both the IMM and OMM by binding to cardiolipin, resulting in the release of mtDNA and mitochondrial proteins [163, 166]. This process is critically dependent on ROS-induced cardiolipin externalization [163]. Compared to GSDMD, GSDMA demonstrates slower and less pronounced accumulation at the plasma membrane, yet exhibits a stronger affinity for mitochondrial targeting [164]. Notably, GSDMA activation induces a dual cell death response: apoptosis, mediated by Cyt c release following mitochondrial injury, and pyroptosis, resulting from plasma membrane rupture. This dual mechanism likely amplifies inflammatory signaling and ensures the irreversibility of cell death [164]. Similarly, the GSDME-NTD exhibits both pro-apoptotic and pro-pyroptotic functions [165].

### cfDNA in inflammation activation

**Fig. 3:**
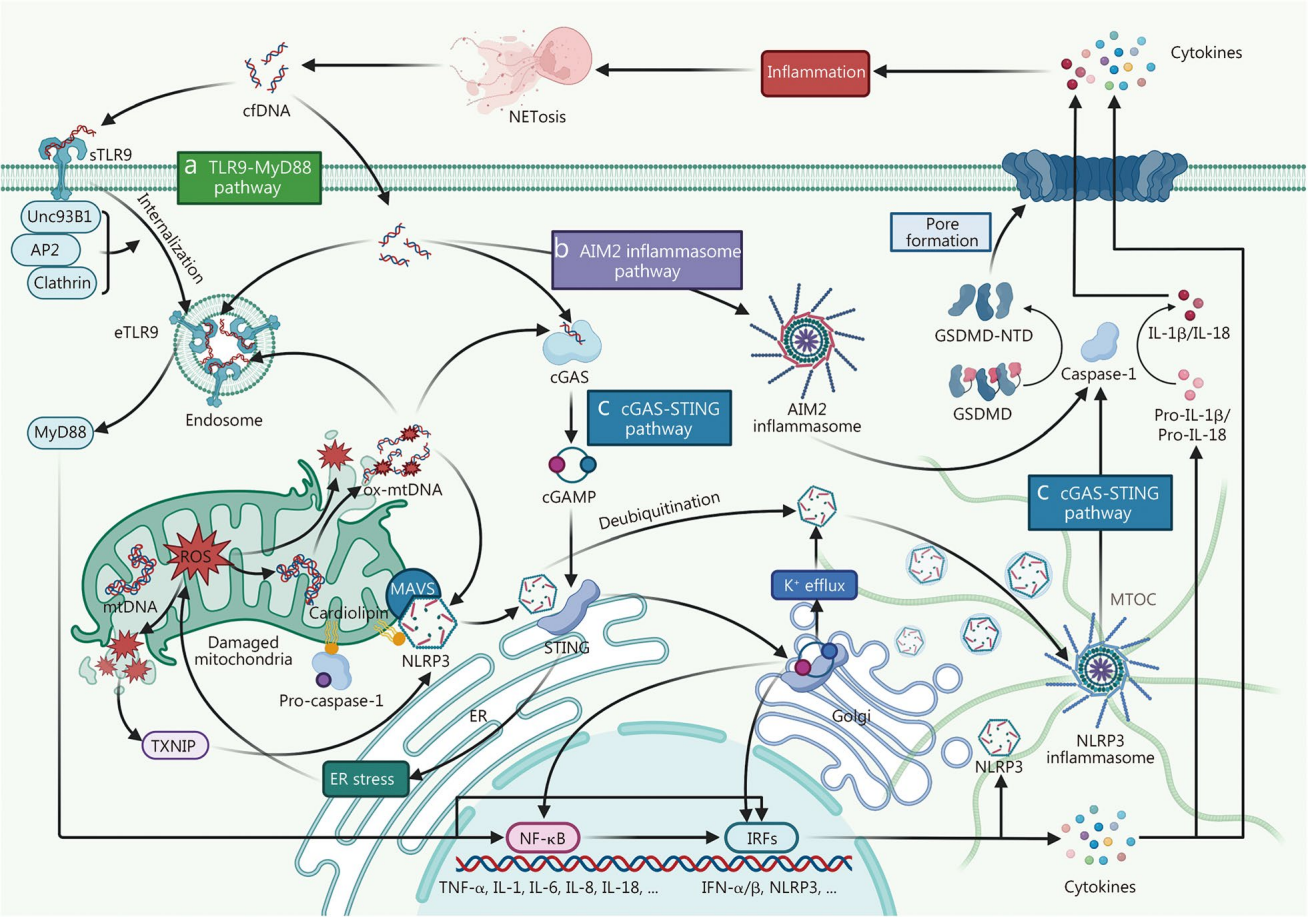
Mechanisms by which cfDNA activates inflammation during sepsis. **a** Toll-like receptor 9 (TLR9)-myeloid differentiation primary response 88 (MyD88) pathway: surface Toll-like receptor 9 (sTLR9) detects cell-free DNA (cfDNA) and, with the aid of UNC-93 homolog B1 (Unc93B1), adaptor protein 2 (AP2), and clathrin, undergoes internalization to form endosomal Toll-like receptor 9 (eTLR9). cfDNA, together with oxidised mitochondrial DNA (ox-mtDNA) released from damaged mitochondria, can also directly activate eTLR9. Activated eTLR9 recruits MyD88, initiating the nuclear factor kappa-B (NF-κB) or interferon regulatory factor (IRF) signaling cascades, which subsequently upregulate a wide range of pro-inflammatory cytokines. **b** Absent in melanoma 2 (AIM2) inflammasome pathway: AIM2 detects cytoplasmic cfDNA, mainly double-stranded DNA (dsDNA), through its HIN200 domain, thereby driving the assembly and activation of the AIM2 inflammasome. The activated AIM2 inflammasome activates pro-caspase-1, which then cleaves gasdermin D (GSDMD) and pro-interleukin (IL)-1β/pro-IL-18. The N-terminal domain of gasdermin D (GSDMD-NTD) inserts into the plasma membrane to form pores, enabling the release of IL-1β, IL-18, and other cytokines, thereby triggering severe inflammation. Notably, the released cytokines generate more cfDNA through NETosis, reinforcing a self-amplifying inflammatory loop. **c** Cyclic GMP-AMP synthase (cGAS)-stimulator of interferon genes (STING) pathway: cfDNA and ox-mtDNA activate cGAS, which catalyzes the production of cyclic GMP-AMP (cGAMP). Subsequently, cGAMP binds to STING, inducing STING dimerization and driving its translocation to the Golgi apparatus. At the Golgi apparatus, STING upregulates the expression of inflammatory factors by activating the NF-κB pathway or the IRF pathway. In addition to the above classical pathways, STING enhances the activation of NOD-like receptor protein 3 (NLRP3) through 4 mechanisms: upregulating NLRP3 expression, mediating NLRP3 deubiquitination, promoting K⁺ efflux, and triggering endoplasmic reticulum (ER) stress. Specifically, ER stress promotes the expression of thioredoxin-interacting protein (TXNIP) and its binding to NLRP3 by inducing excessive production of mitochondrial reactive oxygen species (mtROS), thereby activating NLRP3. Moreover, mitochondrial components such as ox-mtDNA, mitochondrial antiviral signaling protein (MAVS), and cardiolipin also participate in NLRP3 activation. NLRP3 then assembles at the Golgi network and migrates to the microtubule organizing center (MTOC) via the microtubule system. This process promotes the assembly and activation of the NLRP3 inflammasome. The activated NLRP3 inflammasome initiates the same caspase-1 cascade as the AIM2 inflammasome. mtDNA mitochondrial DNA, TNF tumor necrosis factor, IFN interferon

During sepsis, cfDNA primarily triggers inflammatory responses by activating specific PRR pathways, including TLR9-myeloid differentiation primary response 88 (MyD88), AIM2 inflammasome, and cGAS-STING pathways (Fig. 3).

#### TLR9-MyD88 pathway

TLR9 specializes in recognizing unmethylated cytidine-phosphate-guanosine (CpG) motifs commonly found in bacterial and viral genomes, as well as synthetic CpG oligodeoxynucleotides (ODNs) [167, 168]. It is traditionally understood that TLR9 resides within endosomal compartments rather than on the plasma membrane, a localization that serves to limit inappropriate recognition of host DNA and reduce the risk of autoimmune activation [169]. Upon detecting its ligand, TLR9 engages the adaptor protein MyD88 through its Toll/IL-1 receptor (TIR) domain, initiating the assembly of a signaling complex that includes IL-1 receptor-associated kinases 1 and 4 (IRAK1/4) and tumor necrosis factor receptor-associated factor (TRAF) 6 [170]. TRAF6 subsequently activates transforming growth factor-β-activated kinase 1 (TAK1) via ubiquitin-conjugating enzyme 13-dependent K63-linked ubiquitination, which in turn stimulates the NF-κB and mitogen-activated protein kinase pathways, leading to the production and secretion of pro-inflammatory cytokines such as TNF-α, IL-1β, and IL-6 [171]. In plasmacytoid dendritic cells, MyD88 further recruits and activates interferon regulatory factor (IRF) 7, promoting the expression of interferon (IFN)-α/β [172].

Recent studies have revealed that TLR9 is also expressed on the plasma membrane of neutrophils [173], B cells [174], and erythrocytes [175]. To differentiate its 2 localizations, the endosomal variant is referred to as endosomal Toll-like receptor 9 (eTLR9), while the form present on the plasma membrane is designated as surface Toll-like receptor 9 (sTLR9). sTLR9 detects CpG DNA, CpG ODNs, and mtDNA, and is subsequently transported into either IRF- or NF-κB-associated signaling endosomes through the UNC-93 homolog B1-adaptor protein 2-clathrin complex [176–178]. These distinct endosomal pathways then initiate MyD88-dependent signaling, resulting in the production of IFN-α/β and pro-inflammatory cytokines, respectively [178]. Notably, sTLR9 exhibits complex immunomodulatory functions in different cell types. In neutrophils, the sTLR9-positive subset rapidly upregulates IL-10 during the early phase of SIRS, thereby ameliorating local inflammations [179]. In B lymphocytes, sTLR9 acts to suppress activation triggered by CpG ODNs [174]. However, when CpG ODNs are administered as vaccine adjuvants, sTLR9 is internalized and converted into endosomal TLR9, thereby enhancing the humoral immune response by promoting antibody production [180]. In erythrocytes, sTLR9 binds circulating CpG DNA, functioning as a regulatory mechanism to mitigate excessive inflammatory signaling [175]. This protective role, however, comes at a cost: sequestration of CpG DNA leads to the loss of the CD47 “do not-eat-me” signal on erythrocytes, making them susceptible to phagocytosis by red pulp macrophages in the spleen and contributing to the inflammatory milieu characteristic of sepsis [33].

#### AIM2 inflammasome pathway

Inflammasomes are cytosolic multiprotein signaling platforms that detect PAMPs and DAMPs, thereby orchestrating innate immune responses [181]. Their core function is to activate caspases, leading to pyroptosis and the maturation and release of pro-inflammatory cytokines [181]. As a member of the pyrin domain (PYD) and hematopoietic interferon-inducible nuclear proteins domain (HIN)-containing protein family, AIM2 senses double-stranded DNA (dsDNA) of either pathogenic or host origin and drives AIM2 inflammasome assembly and activation [182]. Mechanistically, AIM2 recognises dsDNA through electrostatic interactions within its HIN, inducing a conformational change that dissociates the PYD from the HIN [183]. The multivalent nature of dsDNA permits simultaneous engagement of multiple AIM2 molecules, thereby creating a scaffold for AIM2 oligomerization and inflammasome formation [183]. The PYD of AIM2 forms oligomeric templates through PYD-PYD interactions, recruits apoptosis-associated speck-like protein containing a caspase recruitment domain (ASC), and guides filament formation of the PYD in ASC [184]. The caspase recruitment domain (CARD) of ASC facilitates fibril cross-linking, leading to the formation of compact speck-like aggregates [185]. Structural rearrangements within ASC expose multiple caspase-1 activation sites, thereby creating a robust platform for signal amplification. This arrangement permits a rapid inflammatory response even when only small amounts of PAMPs or DAMPs are present, while also ensuring sufficient cytokine processing before pyroptosis is triggered [185]. Through CARD-mediated interactions, ASC recruits and activates caspase-1, which cleaves pro-IL-1β and pro-IL-18 into their active forms and processes GSDMD to liberate the GSDMD-NTD [186]. The GSDMD-NTD integrates into the plasma membrane, forming pores that drive membrane disruption, pyroptotic cell death, and the subsequent release of cytokines [187]. Importantly, AIM2 inflammasome-mediated cell death demonstrates a DNA concentration-dependent switch: at high DNA levels, caspase-1-driven pyroptosis dominates, whereas at lower DNA concentrations, caspase-8-dependent apoptosis prevails [188]. This bifurcated pathway is essential for preserving immune balance and limiting opportunities for pathogen evasion [188].

#### cGAS-STING pathway

cGAS serves as the principal cytosolic DNA sensor in diverse cell types, recognizing dsDNA of pathogenic or host origin [189]. Upon dsDNA binding, cGAS undergoes a conformational rearrangement in its nucleotidyltransferase domain, enabling the synthesis of the second messenger cGAMP from guanosine triphosphate (GTP) and ATP [190]. cGAMP then binds to STING on the endoplasmic reticulum (ER) membrane, activates STING signaling, and promotes its translocation from the ER to the ER-Golgi intermediate compartment and the Golgi apparatus [191, 192]. Ultimately, activation of the STING-IRF3 and STING-NF-κB pathways induces the expression of IFN-α/β and pro-inflammatory cytokines such as TNF-α, IL-1β, and IL-6 [191, 192]. Interestingly, cGAMP not only functions within the originating cells but can also be transported to neighboring cells through gap junctions or carried by viral particles [190]. Subsequently, cGAMP triggers broader STING activation, coordinating and amplifying the innate immune response [190].

Beyond the classical cGAS-STING-IRF3/-NF-κB axis, the cGAS-STING pathway also triggers inflammasome activation via NLRP3, thereby enabling nucleic acid-sensing inflammatory signaling [193]. Evidence suggests that in human myeloid cells, DNA-induced inflammasome activation predominantly proceeds via the cGAS-STING-NLRP3 axis, with AIM2 playing a non-essential role [194]. Typically, activation of the NLRP3 inflammasome requires 2 distinct signals. The priming step (signal 1) is mediated by DAMPs, PAMPs, or cytokines, leading to the upregulation of inflammasome components [195]. Upon recognition of these priming stimuli, PRRs such as TLRs, IL-1 receptor, and TNF receptor promote rapid induction of NLRP3 or pro-IL-1β through signaling pathways involving MyD88, TIR-domain-containing adapter-inducing interferon-β (TRIF), or other NF-κB-dependent mechanisms [196]. In addition, the priming signal modulates post-translational modifications of NLRP3, including ubiquitination, deubiquitination, and phosphorylation, thereby maintaining the protein in an auto-inhibited yet activation-ready state [197]. Following the priming phase, NLRP3 requires an additional activation signal (signal 2) to initiate inflammasome assembly and activation. Candidate signals include intracellular K^+^ efflux, oxidative stress, mitochondrial damage, and cytosolic release of DNA [195]. STING promotes NLRP3 inflammasome activation through multiple mechanisms during both priming and activation phases [193]. First, STING upregulates NLRP3 expression by activating IRF3 and promoting its nuclear translocation [198]. Second, STING recruits NLRP3 to the ER and relieves its self-repressed state via deubiquitination, thereby enhancing NLRP3 activity and facilitating inflammasome assembly [199]. Furthermore, detection of cytosolic DNA via the cGAS-STING pathway initiates the lysosomal cell death program, resulting in K^+^ efflux and subsequent activation of NLRP3 [194]. STING also promotes excessive production of mtROS through the induction of ER stress. mtROS, in turn, enhances the expression of thioredoxin-interacting protein (TXNIP) and facilitates its interaction with NLRP3, thereby contributing to inflammasome activation [198]. Several studies have established the involvement of the cGAS-STING-NLRP3 axis in NLRP3 inflammasome activation during sepsis, with implications in cardiac, pulmonary, and renal injury associated with the condition [39, 198, 200]. Importantly, oxidised mitochondrial DNA (ox-mtDNA) is essential for NLRP3 activation, it activates the cGAS-STING pathway and may also directly interact with NLRP3 [201–203]. ox-mtDNA is generated under TLR signaling and subsequently cleaved into small fragments by the flap structure-specific endonuclease 1 (FEN1) [156, 203]. In the context of oxidative stress or tissue injury, these small ox-mtDNA fragments escape into the cytosol via mPTP- and VDAC-dependent channels [156, 201, 204].

Beyond the cGAS-STING-NLRP3 axis, the assembly and activation of the NLRP3 inflammasome also depend on coordinated regulation by various organelles and the cytoskeletal system. Several mitochondrial proteins and lipids participate in the spatial clustering and activation of NLRP3. MFN2 recruits NLRP3 to mitochondria and facilitates its oligomerization [205]. By interacting with mitochondrial antiviral signaling protein (MAVS), MFN2 amplifies MAVS-dependent inflammasome activation [205]. MAVS subsequently recruits TRAF3 to ASC and promotes ASC ubiquitylation [206]. Cardiolipin not only provides a platform for NLRP3 recruitment and assembly but also directly activates both NLRP3 and caspase-1 [207]. The Golgi apparatus also represents a critical site for NLRP3 assembly and activation. Dispersed trans-Golgi network membranes attract NLRP3 via phosphatidylinositol-4-phosphate and act as scaffolds that cluster NLRP3 into puncta, thereby promoting ASC aggregation [208]. Additionally, protein kinase D phosphorylates NLRP3, facilitating its relocation from mitochondria-associated ER membranes to the Golgi apparatus [209]. From there, the nascent inflammasome is shuttled along the microtubule network toward the microtubule organizing center, where it clusters into a larger signaling hub to initiate inflammatory responses [210]. Following activation, NLRP3 binds to the PYD of ASC, promoting ASC filament formation. Through its CARD, ASC then recruits pro-caspase-1 and enables its proteolytic activation. The active caspase-1 enzyme subsequently cleaves pro-IL-1β and pro-IL-18 to generate their mature cytokines and processes GSDMD. The liberated GSDMD-NTD inserts into the plasma membrane, creating pores that lead to pyroptotic cell death and the extracellular release of inflammatory mediators [211].

### cfDNA in thrombus formation

**Fig. 4:**
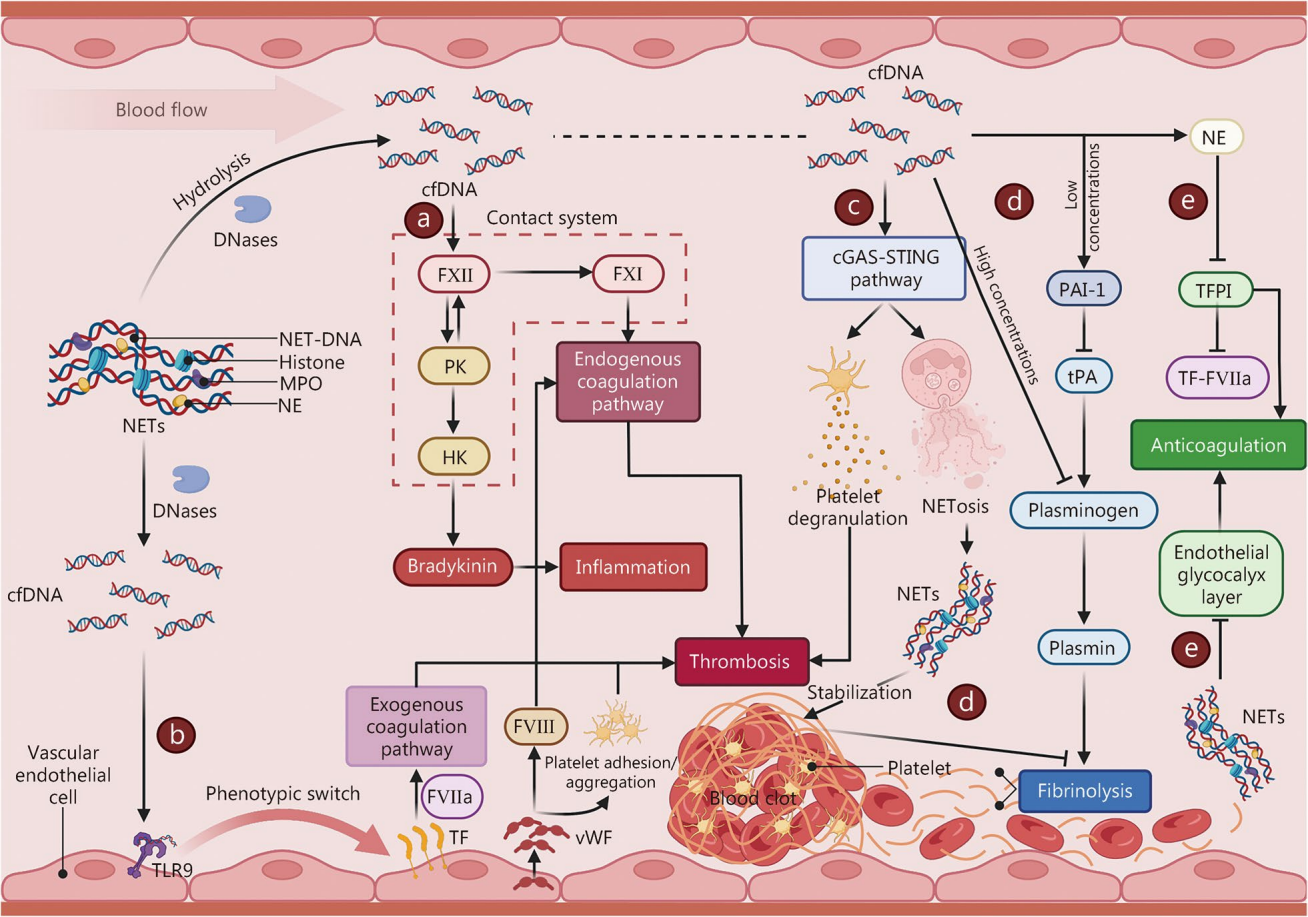
Mechanisms by which cfDNA promotes thrombosis during sepsis. **a** Activation of the contact system: owing to its negative charge, cell-free DNA (cfDNA) adsorbs coagulation factor XII (FXII) and accelerates its auto-activation to activated factor XII (FXIIa). FXIIa then converts prekallikrein (PK) into kallikrein, which cleaves high-molecular-weight kininogen (HK) to release the potent inflammatory mediator bradykinin. Furthermore, PK promotes positive feedback activation of FXII. Once FXIIa accumulates beyond a threshold, it activates factor XI (FXI), thereby initiating the intrinsic coagulation pathway. **b** Endothelial switch to a pro-coagulant phenotype: by engaging Toll-like receptor 9 (TLR9), cfDNA drives endothelial cells to upregulate tissue factor (TF) and massively secrete von Willebrand factor (vWF). TF complexed with activated factor VII (FVIIa) rapidly triggers the extrinsic coagulation pathway, while vWF both promotes platelet adhesion/aggregation and protects factor VIII (FVIII) from proteolytic degradation, indirectly strengthening the intrinsic pathway. **c** Platelet activation and NETosis: through activation of the cyclic GMP-AMP synthase (cGAS)-stimulator of interferon genes (STING) axis, cfDNA may enhance platelet granule release and induce NETosis, collectively accelerating thrombus formation. **d** Suppression of fibrinolysis: cfDNA inhibits fibrinolysis via 2 distinct mechanisms. At physiological low concentrations, cfDNA upregulates plasminogen activator inhibitor-1 (PAI-1), which neutralises tissue-type plasminogen activator (tPA) and thereby indirectly blocks plasmin generation. In sepsis, high concentrations of cfDNA directly compete for plasminogen binding, preventing its activation. In addition, DNA-histone complexes within neutrophil extracellular traps (NETs) reinforce fibrin architecture, rendering clots more resistant to lysis. **e** Impairment of natural anticoagulant pathways: the negatively charged surface of cfDNA facilitates neutrophil elastase (NE)-mediated degradation of tissue factor pathway inhibitor (TFPI), diminishing TFPI’s restraint on the TF-FVIIa complex. DNA-histone complexes also damage the endothelial glycocalyx, causing extensive loss of anticoagulant molecules such as heparan sulphate and antithrombin. NET-DNA neutrophil extracellular trap-derived DNA, MPO myeloperoxidase, DNases deoxyribonucleases

Coagulation disturbances and extensive microvascular thrombosis are hallmarks of severe sepsis and are strongly linked to both elevated short-term mortality and long-term functional decline [7, 212]. Emerging evidence indicates that cfDNA plays a pivotal role in sepsis-associated thrombosis, primarily through mechanisms involving activation of the coagulation cascade, impairment of endogenous anticoagulant pathways, and inhibition of fibrinolytic activity [27, 28] (Fig. 4).

#### Coagulation activation

In septic patients, circulating cfDNA levels are positively associated with thrombin generation, implying a potential procoagulant function of cfDNA [46]. Studies indicate that cfDNA promotes coagulation largely through activation of the contact system and induction of endothelial phenotypic changes [22, 47, 213].

The contact system, comprising factor XI (FXI), factor XII (FXII), prekallikrein (PK), and high-molecular-weight kininogen (HK), plays a dual role in pathological thrombosis and host defence [214]. This cascade is initiated when FXII undergoes autoactivation to activated factor XII (FXIIa) upon encountering negatively charged surfaces. FXIIa then activates PK, which cleaves HK to release bradykinin, a potent mediator of inflammation [215]. Reciprocally, PK accelerates FXII activation, creating a positive feedback loop. Once this cycle surpasses a critical threshold, FXIIa activates FXI, thereby initiating the intrinsic coagulation cascade [215]. Because of its dense negative charge and polyanionic character, cfDNA provides an efficient “foreign surface” that promotes the recruitment and activation of contact system proteins [216]. In particular, cfDNA exhibits strong binding affinity for FXII, FXI, and HK, thereby driving intrinsic pathway activation [47, 213]. Structural investigations further reveal that DNA with hairpin conformations displays enhanced HK-binding capacity and increased plasma stability, features closely linked to elevated procoagulant activity [216]. Furthermore, LMW DNA and single-stranded DNA demonstrate stronger procoagulant effects, as they expose more single-strand ends capable of forming hairpins [22].

In sepsis, endothelial cells shift toward a procoagulant phenotype, characterized by elevated TF expression and extensive secretion of vWF [217]. TF serves as the central initiator of the extrinsic coagulation pathway, rapidly activating clot formation upon binding to FVIIa [218]. Concurrently, vWF supports platelet adhesion and aggregation while stabilizing factor VIII (FVIII) against proteolytic degradation, thereby reinforcing intrinsic coagulation [219]. Experimental study has shown that cfDNA stimulates endothelial cells through TLR9, driving TF expression and vWF release, which in turn enhances platelet adhesion and potentiates both extrinsic and intrinsic pathways of coagulation [22]. In addition, the cGAMP-STING signaling axis has been implicated in promoting platelet granule release and inducing NETosis, further accelerating thrombus development during sepsis. cfDNA, as a classic activator of the cGAS-STING pathway, may be involved in this process [48, 190].

#### Inhibition of anticoagulation and fibrinolysis

Suppression of the fibrinolytic system is considered a key mechanism by which sepsis leads to DIC and organ dysfunction [220]. A study has shown that the effect of cfDNA on fibrinolysis is concentration-dependent. At physiological (low) concentrations, cfDNA significantly enhances plasminogen activation by tissue-type plasminogen activator (tPA) through a templating mechanism [50]. It also facilitates the suppression of tPA activity by plasminogen activator inhibitor-1 (PAI-1), thereby dynamically balancing fibrin generation and breakdown [50]. In contrast, the markedly increased plasma cfDNA levels observed in sepsis predominantly exert antifibrinolytic actions [27]. At high concentrations, cfDNA may either compete with fibrin for plasminogen binding or simultaneously associate with both fibrin and plasminogen to form inactive ternary complexes, thereby impairing plasmin-dependent fibrin degradation [27, 50]. Elevated cfDNA further promotes the formation of denser, more compact fibrin clots that are resistant to lysis by plasmin [27]. DNA-histone complexes amplify these effects by enlarging fibrin fibre diameter, enhancing the mechanical robustness of the fibrin matrix, and binding fibrin degradation products, thus generating a fibrinolysis-resistant scaffold [49].

In addition to these antifibrinolytic roles, cfDNA profoundly disrupts anticoagulant pathways. Its negatively charged surface accelerates NE-mediated inactivation of TFPI, diminishing TFPI’s ability to inhibit both the TF-FVIIa complex and the prothrombinase complex [51, 56]. Furthermore, DNA-histone complex, in synergy with proteases and inflammatory mediators, degrades the endothelial glycocalyx, resulting in significant loss of anticoagulant molecules such as heparan sulphate and antithrombin [28].

### cfDNA in sepsis-associated organ injury

In patients with sepsis, especially those with non-pulmonary infection sources, plasma cf-mtDNA levels measured after 48 h are significantly and positively correlated with the occurrence of acute respiratory distress syndrome [221]. Similarly, in mice with lipopolysaccharide (LPS)-induced ALI, cfDNA levels in serum and bronchoalveolar lavage fluid are significantly increased and positively correlated with lung injury scores [38]. Moreover, intraperitoneal administration of purified mtDNA into healthy mice reproduces the pathological characteristics observed in SA-ALI [222]. These findings suggest that cfDNA serves as a significant pathogenic contributor to SA-ALI. At the mechanistic level, the cGAS-STING signaling pathway plays a central role in cfDNA-induced SA-ALI. Evidence has shown that lung damage is notably reduced in STING-deficient mice compared to wild-type counterparts following mtDNA injection [223]. In sepsis models, cfDNA promotes macrophage activation through the cGAS-STING pathway, resulting in the production of pro-inflammatory cytokines and recruitment of additional immune cells, which together contribute to inflammatory cell infiltration and apoptosis within lung tissue [38]. Meanwhile, cfDNA induces autophagy dysfunction (particularly impaired lysosomal acidification) by activating the cGAS-STING pathway in macrophages, further contributing to SA-ALI [223]. Moreover, the cfDNA-cGAS-STING-NLRP3 axis is also involved in SA-ALI pathogenesis [39]. Notably, STING promotes the interaction between DRP1 and GSDMD-NTD in SA-ALI, triggering mitochondrial fission and mtDNA release [224]. These findings suggest that the cGAS-STING pathway not only represents the key mechanism for cfDNA-induced SA-ALI but also acts as an amplifier that promotes additional cfDNA production. In addition, although fewer studies exist, current evidence also implicates the TLR9-MyD88 pathway and the AIM2 inflammasome in cfDNA-induced SA-ALI [30, 31, 37].

Similar to SA-ALI, injecting mitochondrial fragments rich in mtDNA into the veins of healthy mice can mimic the pathological manifestations of SA-AKI. Pre-degrading mtDNA in mitochondrial fragments with deoxyribonucleases (DNases) suppresses these pathological effects [32]. Studies have shown that cf-mtDNA promotes inflammatory factor release and mitochondrial dysfunction in renal tubules during sepsis by activating the TLR9 signaling pathway in immune cells, particularly dendritic cells and B cells [32, 34]. Specifically, TLR9-activated bone marrow-derived dendritic cells induce γδT cells to secrete IL-17A via IL-23 production, thereby promoting SA-AKI development [34]. In addition, in SA-AKI mouse and cell models, mtDNA has been observed to induce inflammatory factor release and pyroptosis by activating the cGAS-STING-NLRP3 axis in renal tubular epithelial cells [40]. Research by Cao et al. [198] further demonstrated that STING activates the NLRP3 inflammasome through a dual mechanism, ultimately promoting inflammation, pyroptosis, and apoptosis in renal tubular cells. On one hand, STING directly upregulates NLRP3 expression via the IRF3 pathway. On the other hand, STING triggers excessive mtROS generation by inducing ER stress, thereby promoting TXNIP binding to NLRP3 and activating NLRP3. Notably, although the AIM2 inflammasome plays an important role in cfDNA recognition, its pathogenic role in SA-AKI has not been fully characterized. A possible explanation is that most current studies employ relatively short mtDNA fragments (17 kb) as inducers, which cannot reach the minimum length required for AIM2 recognition (80 kb) [225].

Compared with SA-ALI and SA-AKI, fewer studies have investigated cfDNA in other sepsis-associated organ injuries. Li et al. [200] reported that LPS stimulation activates the STING-IRF3-NLRP3 pathway in cardiomyocytes, inducing inflammatory responses, apoptosis, and pyroptosis. Zhang et al. [41] found that Kupffer cells promote mtDNA release through DRP1 and activate STING signaling during sepsis, leading to massive inflammatory factor production and hepatocyte damage. Hu et al. [42] revealed that the mtDNA-STING pathway promotes intestinal epithelial cell apoptosis and disrupts intestinal barrier function during sepsis, ultimately exacerbating bacterial translocation, systemic inflammation, and multi-organ injury. In addition, Zeng et al. [43] found that mtDNA induced microglial pyroptosis and amplified neuroinflammation through activating the cGAS-STING-NLRP3 inflammasome pathway in cecal ligation and puncture (CLP) mice, thereby promoting the progression of SAE. Although research in SAE remains limited, accumulated evidence suggests that cfDNA-induced neuroinflammation is an important mechanism driving ischemic brain injury and neurodegeneration [226, 227]. cfDNA initiates a neuroinflammatory cascade by activating cytoplasmic DNA sensors, particularly cGAS and NLRP3 inflammasome, thereby exacerbating neurological lesions [228]. It has been reported that ox-mtDNA directly binds to NLRP3, triggering its activation and mediating brain injury in ischemic stroke [227]. Similarly, mtDNA is involved in sevoflurane-induced postoperative cognitive dysfunction in mice through activation of the cGAS-STING-NLRP3 pathway [229]. The mtDNA-cGAS-STING pathway also contributes to the pathogenesis of neurodegenerative diseases such as Parkinson’s disease, Alzheimer’s disease, and Huntington’s disease [230]. The critical pathological role of cfDNA in neuroinflammation may help explain the occurrence of SAE and long-term cognitive dysfunction in sepsis survivors. Overall, current research on cfDNA-induced sepsis-associated organ injury is most comprehensive in the lungs, with the mtDNA-cGAS-STING axis being the most extensively characterized pathway. However, studies on the kidneys, heart, liver, intestines, and brain remain scarce, and the role of nDNA has not been fully elucidated. Future investigations should expand to additional organs and conduct systematic comparisons of the differential effects of mtDNA and nDNA to complete the mapping of cfDNA-mediated multi-organ damage.

## cfDNA in sepsis monitoring

### Sepsis monitoring in the era of liquid biopsy

Liquid biopsy is a minimally invasive and dynamic approach for disease monitoring, relying on the detection of cell-free nucleic acids (cfDNA and cfRNA) in body fluids [231]. As early as 1994, cfDNA was reported to have clinical utility in tumor diagnosis, assessment of therapeutic response, and prognostic evaluation [232]. With the rapid advancement of NGS, cfDNA analysis has achieved major improvements in sequencing speed, depth, and throughput [233]. These technological developments have greatly expanded the clinical applications of cfDNA in oncology, organ transplantation, infectious diseases, and prenatal testing, marking the emergence of the liquid-biopsy era [231].

#### cfDNA in the diagnosis and prognosis of sepsis

Clinical research on cfDNA as a biomarker for sepsis monitoring was initiated in 2006. The initial report showed that plasma cfDNA concentrations were markedly elevated in septic patients and exhibited a positive correlation with both disease severity and mortality risk [57]. In a subsequent large-scale study, Saukkonen et al. [234] evaluated the prognostic significance of cfDNA in patients with severe sepsis and septic shock, demonstrating that cfDNA levels served as an independent predictor of ICU mortality and were strongly associated with disease severity and the development of organ dysfunction. Moreover, longitudinal analyses conducted by Dennhardt et al. [59] in sepsis survivors revealed that cfDNA levels were substantially elevated during the early phase of illness and progressively declined to baseline as recovery ensued, underscoring its value for early diagnosis. Furthermore, several systematic reviews evaluated the diagnostic and prognostic performance of cfDNA in sepsis, all of which supported its clinical utility [235, 236]. A meta-analysis conducted in 2023, comprising 18 studies, revealed that plasma levels of cfDNA were markedly higher in septic patients compared to healthy individuals and were significantly increased in non-survivors relative to survivors. The findings further suggested that cfDNA serves as an early and specific biomarker for identifying sepsis and predicting mortality shortly after hospital admission or the onset of illness [235]. Consistent results were reported in a subsequent 2024 meta-analysis involving 2950 participants. In this study, pooled analysis showed that cfDNA demonstrated high sensitivity (0.81 for diagnosis; 0.78 for prognosis) and high specificity (0.87 for diagnosis; 0.78 for prognosis) for both the diagnosis and prognosis of sepsis [236].

In addition, the correlation between cfDNA and systemic inflammation, as well as sepsis severity, has been validated in numerous studies. Plasma cfDNA levels in septic patients exhibit a positive correlation with inflammatory markers such as procalcitonin (PCT), C-reactive protein (CRP), and IL-6, consistent with the pro-inflammatory effects of cfDNA revealed by basic research [237–239]. Plasma cfDNA levels also increase with disease severity and show a positive correlation with clinically established severity scoring systems, such as Acute Physiology and Chronic Health Evaluation II (APACHE II) and Sequential Organ Failure Assessment (SOFA) scores [20, 61, 237, 240]. These findings not only support cfDNA as a marker for evaluating sepsis severity but also highlight its potential in monitoring therapeutic response. In a reported case of septic shock, despite marked reductions in white blood cell count and PCT levels following continuous renal replacement therapy (RRT) and extracorporeal membrane oxygenation, cfDNA levels remained persistently elevated. The patient ultimately died from septic shock on hospital day 8, indicating the heightened sensitivity of cfDNA in reflecting treatment response and disease progression [241]. Additionally, cfDNA may serve as a predictor of specific therapeutic needs and treatment outcomes in septic patients. Evidence has demonstrated significantly higher cfDNA levels in patients requiring RRT, suggesting its value as a biomarker for anticipating RRT necessity. Among cfDNA components, the cytochrome C oxidase subunit III fragment appears to contribute most strongly to predicting RRT requirement, pointing to heterogeneity among cfDNA subtypes in sepsis monitoring [59]. Furthermore, a recent secondary analysis of septic shock cases suggested that therapeutic plasma exchange may offer particular benefit to patients with increasing cfDNA levels [242]. Collectively, these studies demonstrate the potential applications of cfDNA in guiding sepsis therapy, highlighting its promise as a biomarker for precision medicine in sepsis.

Despite the observed correlations, cfDNA may offer distinct advantages over conventional inflammatory biomarkers and clinical scoring systems in sepsis diagnosis and prognosis. PCT is not only the most extensively studied sepsis biomarker but is also regarded as a therapeutic target owing to its toxic properties [243–245]. PCT levels correlate with disease severity and are widely used to monitor treatment response and guide antibiotic initiation and discontinuation [243]. However, cfDNA has demonstrated diagnostic accuracy comparable to, or surpassing, that of PCT and offers additional strengths in risk stratification and prognostic evaluation [61, 240]. Similarly, CRP, a long-established acute-phase reactant employed as a non-specific indicator of infection and inflammation [245], is less effective for sepsis assessment compared with cfDNA. Evidence indicates that cfDNA outperforms CRP in both diagnostic and prognostic contexts [61, 246]. Moreover, cfDNA exhibits significantly greater predictive power than clinical scoring systems such as the MODS and APACHE II scores, as well as biomarkers including IL-6, protein C, and thrombin-antithrombin complexes, in forecasting ICU mortality among septic patients [58]. Notably, substantial evidence suggests that integrating cfDNA into predictive models incorporating clinical scoring systems (such as MODS, APACHE II, SOFA, and quick SOFA) or other biomarkers (such as PCT and protein C) can further enhance diagnostic and prognostic capability [20, 58, 247, 248]. Additionally, a growing number of candidate sepsis biomarkers, such as calcitonin gene-related peptide [249], CD38^high^ monocytes [250], and exosomes [251], are demonstrating potential in sepsis diagnosis and treatment. Therefore, future research should further evaluate and optimize the performance of cfDNA-integrated predictive models. Concurrently, comparative analyses and combined applications of cfDNA with these novel sepsis markers should be advanced.

#### cfDNA in organ-specific functional monitoring of sepsis

Early studies established a strong correlation between cfDNA levels and the development of MODS in sepsis [57, 234]. However, cfDNA levels appeared to provide only a coarse reflection of overall organ function rather than enabling precise monitoring of specific organ functions. Recent progress in the analysis of cfDNA methylation profiles has enabled novel approaches for organ-specific functional monitoring [62]. Evidence has demonstrated that individual cell types possess unique and stable DNA methylation signatures essential for maintaining tissue-specific gene expression patterns [252]. The high degree of inter-individual consistency observed in cfDNA methylation profiles renders them suitable biomarkers for determining the tissue of origin [252].

Utilizing this characteristic, organ-specific cfDNA methylation markers, such as those corresponding to the heart, liver, and kidney, have been developed to support targeted monitoring of sepsis-associated organ injury. In the context of myocardial injury monitoring, Zemmour et al. [253] analyzed tissue-specific methylation profiles and identified the family with sequence similarity 101, member A (FAM101A) locus as a cardiomyocyte-specific cfDNA marker. Myocardial cfDNA exhibited high specificity and sensitivity for detecting cardiomyocyte death, with dynamic fluctuations that closely reflected disease progression. Notably, unlike troponin, myocardial cfDNA was not influenced by delayed biomarker clearance due to hepatic or renal dysfunction, thereby offering more reliable diagnostic accuracy [253]. Similarly, Lehmann-Werman et al. [254] employed liver-specific methylation markers to track liver injury. Their findings showed that liver-derived cfDNA outperformed conventional liver function biomarkers in both sensitivity and specificity, as aspartate aminotransferase and alanine aminotransferase may originate from muscle or cardiac tissues. Additionally, the shorter half-life of liver-specific cfDNA allowed a more rapid reflection of liver injury dynamics [254]. Moreover, cfDNA methylation markers specific to renal epithelial and endothelial cells have recently been characterized, offering early, accurate, and dynamic monitoring of SA-AKI [255]. Based on the research above, organ-specific cfDNA methylation markers demonstrate unique advantages over conventional biomarkers in terms of specificity, sensitivity, and dynamic monitoring capabilities. However, current studies remain limited in both number and scale. Future work should focus on conducting larger prospective clinical trials to validate their efficacy, while establishing standardized protocols for sample processing and analytical workflows to ensure result comparability and reliability. Furthermore, exploration of other promising cfDNA epigenetic markers for monitoring organ function is warranted.

#### cfDNA in pathogen detection of sepsis

In sepsis monitoring, most applications of cfDNA remain at the clinical research stage. However, in pathogen detection, mcfDNA sequencing (mcfDNA-seq) has taken the lead in achieving clinical laboratory-level transformation [19]. mcfDNA-seq is an mNGS approach that identifies pathogens by high-throughput sequencing and bioinformatic analysis of mcfDNA from patient body fluids [19, 256]. Compared with blood culture, mcfDNA-seq offers substantial improvements in both diagnostic timeliness and sensitivity [19].

The median turnaround time from sample collection to reporting for mcfDNA-seq is estimated at 48 h, including transportation [257, 258]. Since testing is typically conducted in third-party laboratories, there remains considerable potential to accelerate the workflow. Excluding transport, the assay itself can be completed in about 30 h, thereby providing critically ill patients with valuable additional time for timely diagnosis and treatment [65]. Traditional antibiotic susceptibility testing and resistance gene analysis require prior pathogen isolation, which contributes to diagnostic delays. By contrast, mcfDNA-seq simultaneously identifies pathogens and detects resistance genes, substantially improving diagnostic efficiency [259]. Notably, mcfDNA-seq is capable of detecting nearly 75% of causative pathogens up to 72 h before clinical symptom onset, underscoring its value for early sepsis detection and intervention [260].

In terms of sensitivity, mcfDNA-seq not only identifies a broader spectrum of pathogens but also effectively detects microorganisms in blood culture-negative samples [65, 258, 261]. Studies have demonstrated that mcfDNA-seq detects bacteria, fungi, viruses, and parasites, and is highly effective for identifying rare or low-abundance microbes missed by conventional methods [258, 259]. Several clinical case reports have demonstrated the successful use of mcfDNA-seq in identifying rare pathogens, including Leptospira santarosai [262], Naegleria fowleri [263], *Mycobacterium tuberculosis* [264], Taenia solium [265], and West Nile virus [266]. Beyond known pathogens, mcfDNA-seq has facilitated the discovery of previously unrecognized agents such as *Psychrobacter* sp. 310 [267], *Basidiobolus meristosporus* [268], and Cache Valley virus [269], thereby broadening the spectrum of infectious diseases. Importantly, a single mcfDNA-seq assay is capable of detecting multiple pathogens concurrently, making it particularly valuable for diagnosing polymicrobial infections in immunocompromised patients [270]. Furthermore, because mcfDNA-seq detects mcfDNA rather than viable organisms, its diagnostic performance is less affected by prior antibiotic administration. Multiple studies have shown that mcfDNA-seq maintains a high positive rate under antibiotic exposure, significantly superior to blood culture [271–273].

Currently, several commercial mcfDNA-seq platforms have been developed, further promoting clinical application. For example, the Karius test currently covers 1250 clinically relevant pathogens and has demonstrated excellent sensitivity and specificity in rigorous clinical and analytical validations [274]. Additionally, the platform provides results as early as the day after sample receipt, offering critical time for early diagnosis and targeted treatment of sepsis and other life-threatening infections [274]. With the ongoing decline in sequencing expenses and continuous improvements in bioinformatics algorithms, mcfDNA-based pathogen identification is expected to emerge as a standard approach for the diagnosis and management of sepsis.

### Future directions for next-generation liquid biopsy technologies

As an innovative diagnostic strategy, cfDNA-based liquid biopsy has quickly become a central focus in sepsis monitoring research. Current efforts are directed toward overcoming technical limitations by improving sequencing accuracy, refining data-analysis algorithms, and enhancing detector performance, thereby advancing the development of next-generation liquid-biopsy platforms. These emerging technologies hold the potential to drive a paradigm shift in the monitoring and management of sepsis.

#### Third-generation sequencing (TGS)

Despite the widespread application of NGS in both research and clinical practice, its utility is limited by 2 major technical challenges. First, the relatively short read lengths (about 35–300 bp) hinder accurate resolution of long repetitive elements and large structural variants, thereby increasing the risk of genome assembly errors [275]. Second, the polymerase chain reaction (PCR), an essential component of NGS workflows, inevitably introduces amplification bias. Such bias compromises sequencing-coverage uniformity and may result in misinterpretation of genetic variants or microbial community composition [276, 277]. As a result, NGS typically requires greater sequencing depth to maintain accuracy, which in turn generates vast amounts of data, escalating both computational demands and the complexity of bioinformatic analyses [278].

In this context, TGS has emerged as a transformative technology, representing a third revolution in sequencing through its single-molecule real-time approach and ultra-long read lengths [279]. Unlike NGS, single-molecule sequencing eliminates the need for PCR amplification, thereby reducing GC bias and improving resolution in GC-rich genomic regions [280]. Moreover, it enables direct detection of base modifications on native DNA strands, such as methylation, providing a powerful platform for epigenetic studies [281]. Furthermore, real-time sequencing technology detects base incorporation by directly observing fluorescent signals, enabling simultaneous analysis of polymerase kinetics and high-precision nucleic acid sequence output [282]. This approach also avoids the stepwise pause operation of NGS, increasing sequencing-cycle speed by about 4 orders of magnitude [282]. Additionally, the ultra-long read lengths of TGS allow it to span long repetitive sequences and structural variation regions, significantly enhancing genome assembly contiguity and variant detection accuracy [283].

TGS has demonstrated exceptional performance in both pathogen detection and epigenetic research [284]. Nanopore sequencing, for example, achieves real-time identification of pathogens and simultaneous analysis of drug-resistance genes [285, 286]. Moreover, the entire process from sample preparation to identification reporting takes only about 4–8 h, far surpassing traditional NGS solutions [287]. Notably, portable nanopore sequencing devices offer high-throughput sequencing capability in field or resource-constrained settings, further broadening the scope of TGS applications [288]. Thus, TGS is spearheading advances in liquid biopsy and sepsis monitoring.

#### Machine learning (ML)

Benefiting from the rapid advancement of high-throughput sequencing technology, the sequencing depth and coverage of cfDNA have been significantly enhanced, generating multidimensional cfDNA datasets encompassing genomics, epigenomics, transcriptomics, and microbiomics [289]. This expansion shifts cfDNA analysis targets from single quantitative signals such as point mutations to multidimensional continuous signals, including fragment-size distributions and epigenetic modifications, surpassing the processing capabilities of traditional analytical methods [290]. Faced with the dual challenges of high dimensionality and complexity, ML, with its efficient algorithmic frameworks and self-optimization mechanisms, has become an essential tool for in-depth multi-omics analysis of cfDNA [290]. As a core branch of artificial intelligence, ML parses data through algorithms, constructs statistical models, and enables autonomous optimization based on experience, thereby facilitating automated feature extraction and prediction from cfDNA datasets [291]. Classical algorithms such as logistic regression and random forests allow models to learn from vast cfDNA data and extract critical information, improving training efficiency and generalizability [292, 293]. Moreover, ML can leverage multi-view learning approaches to integrate multi-omics information and construct high-dimensional feature spaces, further enhancing predictive performance [294, 295]. Additionally, ensemble ML models combining the strengths of various algorithms effectively overcome the performance limitations of individual methods [296].

In sepsis monitoring, ML models based on cfDNA data have achieved notable progress. Chen et al. [297] developed a random-forest model using balanced subsampling of cfDNA sequencing data from 38 sepsis patients and 118 healthy controls. This model enabled rapid diagnosis of sepsis within 24 h, achieving a sensitivity of 0.91 and a specificity of 0.83, while simultaneously identifying most pathogens. Similarly, Jing et al. [298] developed a random-forest model incorporating plasma cfDNA metagenomic features, cfDNA concentration, fragmentation profiles, and microbial composition, obtained from ICU patients, enabling early sepsis diagnosis, mortality prediction, and precise pathogen identification on the first day of admission. In addition, ML offers distinct advantages in monitoring organ function in sepsis due to its effective tissue-of-origin tracing capability. Moss et al. [66] introduced a methylation-based deconvolution algorithm for comprehensive and quantitative assessment of cfDNA tissue sources across various pathological states. Subsequently, a cfDNA decomposition method based on the expectation–maximization algorithm improved the accuracy of tissue origin inference by handling low-coverage and high-noise data, resolving unknown cell types, and autonomously selecting CpG sites [299]. Importantly, the integration of multi-omics cfDNA data with other biomarkers and clinical parameters presents a promising approach for developing advanced sepsis-monitoring systems. Recently, Cheungpasitporn et al. [300] investigated the analytical and predictive potential of ML applied to multidimensional features, including physiological indicators, laboratory results, and medical history, in the clinical management of SA-AKI. Their results showed that ML significantly improved diagnostic and predictive accuracy, providing decision support for personalized treatment plans and revealing a new paradigm for precision sepsis management. However, obstacles including data privacy concerns, limited dataset size, and insufficient model interpretability continue to hinder clinical application [301]. Future research should focus on establishing standardized data governance frameworks, fostering interdisciplinary collaboration, and advancing the development of more transparent and interpretable models to facilitate the effective implementation of ML in cfDNA analysis and clinical decision-making.

#### Wearable nucleic acid testing (NAT) sensors

Wearable biosensors (WBs) are compact electronic devices embedded in skin-adherent platforms such as patches, smartwatches, or textiles [302]. Utilizing advanced biosensing technologies, WBs facilitate continuous and real-time monitoring of physiological indicators and biomarkers, supported by wireless communication systems and intelligent data-processing modules [303]. Within the framework of precision medicine, WBs are evolving rapidly, significantly transforming diagnostics toward portable, individualized, and smart healthcare solutions [302]. Recent advancements in microfluidics and isothermal nucleic acid amplification technologies (iNAATs) have accelerated the development of wearable NAT sensors, thereby advancing cfDNA monitoring capabilities [304]. Microfluidic systems enable efficient manipulation and analysis of fluids within miniaturized architectures [305], while iNAATs eliminate reliance on conventional thermal-cycling devices, simplifying the integration of nucleic acid assays into wearable formats [306]. Driven by these enabling technologies, a range of wearable NAT sensor designs has emerged, demonstrating strong performance in pathogen detection.

For instance, a microneedle-based, patch-type NAT sensor introduced in 2019 achieved minimally invasive, highly sensitive, and rapid detection of Epstein-Barr virus cfDNA in interstitial fluid [307]. In the same year, Kong et al. [308] developed a wristband-format NAT sensor for rapid and on-site detection of human immunodeficiency virus type 1 (HIV-1) DNA. Furthermore, wearable NAT sensors have been incorporated into face masks for detecting severe acute respiratory syndrome coronavirus 2 (SARS-CoV-2) in exhaled aerosols [309]. While current reports remain limited, wearable NAT technologies have already exhibited significant potential for sepsis surveillance. Yang et al. [310] integrated a clustered regularly interspaced short palindromic repeats (CRISPR)-CRISPR-associated protein 9 (Cas9) functionalized graphene biointerface into a wearable microneedle platform, enabling continuous, real-time, and accurate monitoring of interstitial-fluid cfDNA for up to 10 d in patients with sepsis. Building upon this, the team later introduced a tetrahedral nanostructure-NgAgo system, capable of detecting unamplified cfDNA in real time at ultralow concentrations (0.3 fmol/L), with stable functionality maintained over a 14 d period [311]. These wearable NAT systems offer comprehensive and real-time data across the full clinical spectrum of sepsis care, from pre-hospital screening to in-hospital treatment and post-discharge follow-up, through dynamic cfDNA monitoring, potentially reshaping sepsis management practices. Future integration of multiparametric sensing and advanced ML-based analytics may further improve detection precision and support personalized early warning and intervention strategies [312, 313].

## cfDNA in sepsis treatment

**Fig. 5:**
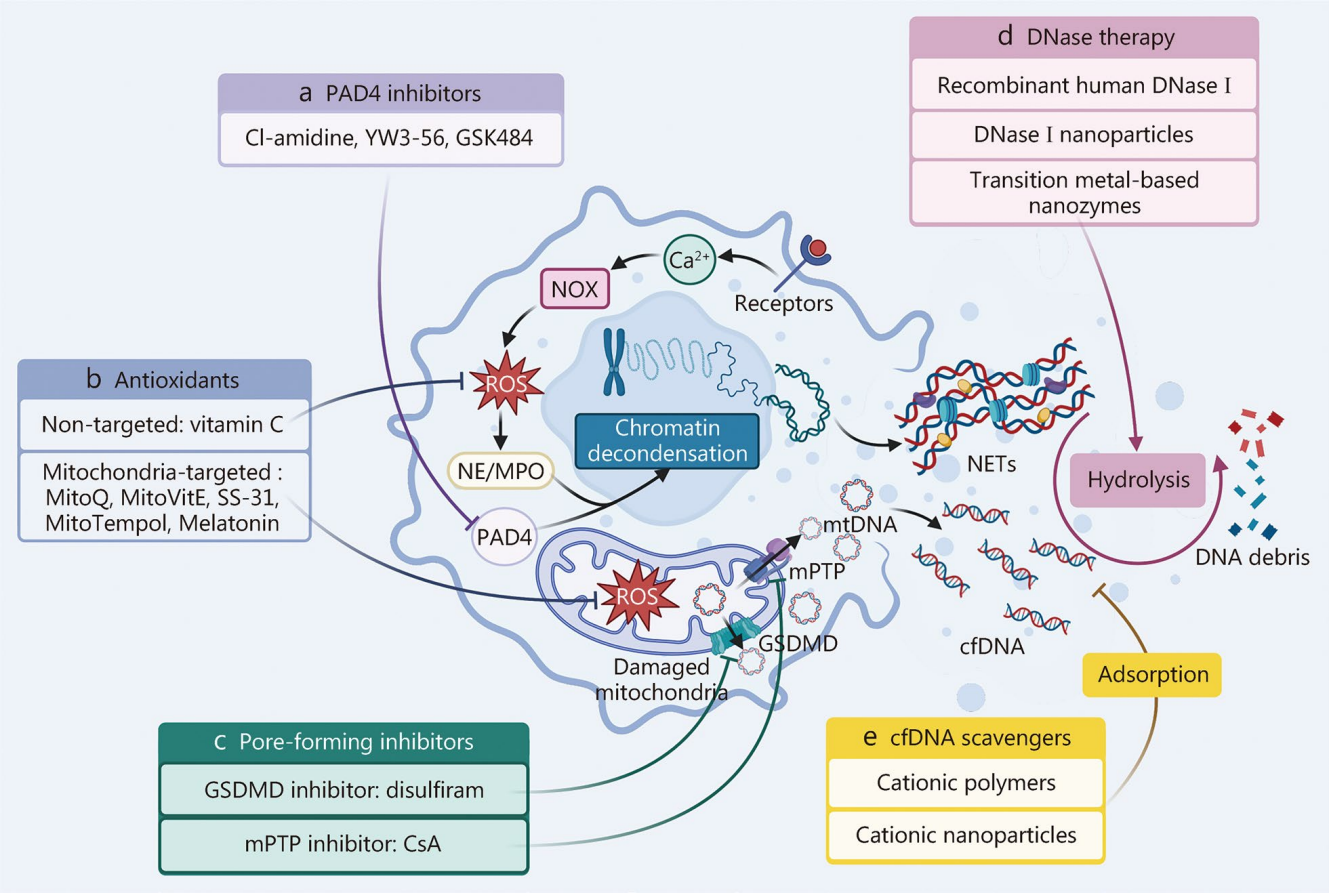
Therapeutic strategies targeting cfDNA in sepsis. **a** Peptidylarginine deiminase 4 (PAD4) inhibitors. Representative compounds such as Cl-amidine, YW3-56, and GSK484 inhibit histone arginine deimination, thereby preventing chromatin decondensation and neutrophil extracellular trap (NET) formation.** b** Antioxidants. Reactive oxygen species (ROS) are critical mediators of NETosis and mitochondrial DNA (mtDNA) release. Both general and mitochondria-targeted antioxidants suppress NETs and mtDNA release by inhibiting ROS generation.** c** Pore-forming inhibitors. Agents such as disulfiram and cyclosporine A (CsA) reduce mtDNA release by inhibiting the formation of mitochondrial membrane pores. **d** Deoxyribonuclease (DNase) therapy. Recombinant human DNase I, DNase I nanoparticles, and transition metal-based nanozymes degrade extracellular NETs and cell-free DNA (cfDNA), thus neutralizing their pathological effects. **e** cfDNA scavengers. Cationic polymers or nanoparticles electrostatically bind cfDNA to form inert complexes, rapidly reducing its pathological activity. GSDMD gasdermin D, mPTP mitochondrial permeability transition pore, NOX nicotinamide adenine dinucleotide phosphate hydrogen oxidase, NE neutrophil elastase, MPO myeloperoxidase

Multiple therapeutic strategies targeting cfDNA have been proposed for the management of sepsis. These approaches can be broadly categorized into 2 types: reducing the release of cfDNA and enhancing its clearance (Fig. 5).

### Reducing cfDNA release

#### PAD4 inhibitors

In *PAD4*-knockout mice, NET formation is absent, and reductions in inflammation, improved organ function, and enhanced survival have been observed during sepsis, supporting the therapeutic relevance of PAD4 inhibition [314–316]. Cl-amidine, a widely studied pan-PAD inhibitor, irreversibly inhibits PAD4 by covalently modifying the Cys645 catalytic residue [317, 318]. Both in vitro and in vivo models have shown that Cl-amidine suppresses LPS-induced NETosis, thereby effectively lowering cfDNA levels [319, 320]. In sepsis models, Cl-amidine demonstrated anti-inflammatory and anticoagulant effects, conferred multi-organ protection, and improved survival outcomes [321–324]. Similarly, YW3-56, a dual PAD2/PAD4 inhibitor, decreased cfDNA concentrations and exerted protective effects in septic mice [325, 326].

However, pan-PAD inhibitors face challenges such as poor isoform specificity, high cytotoxicity, limited in vivo half-life, and narrow therapeutic windows, all of which constrain clinical applicability [317]. Structural modifications have been employed to improve the safety and efficacy profiles of these agents. For instance, BB-Cl-amidine extended the half-life of Cl-amidine from about 15 min to 1.75 h. Although this duration remains short, it highlights a direction for further optimization [327]. Recently, hydroxy-substituted naphthalene- and quinoline-based PAD inhibitors were developed by Jia et al. [328], with compounds 13 and 16 exhibiting potent inhibition (IC_50_ values of 0.273 μmol/L for PAD1 and 0.204 μmol/L for PAD4, respectively) and low cytotoxicity, thereby expanding the therapeutic window. To reduce off-target effects, several PAD4-selective inhibitors have been engineered. GSK199 and GSK484 achieve selective and reversible inhibition through the induction of β-hairpin formation at the active site, minimizing off-target effects and long-term toxicity associated with irreversible inhibitors [329]. Reportedly, GSK199 preserved over 95% cell viability at concentrations up to 20 mmol/L, whereas BB-Cl-amidine demonstrated significant cytotoxicity at concentrations exceeding 1 mmol/L [330]. GSK484 has been shown to lower cfDNA levels and attenuate SA-ALI in preclinical models [331, 332]. Moreover, Ruiz-Hernández et al. [333] identified amodiaquine, folic acid, and pyronaridine as stable PAD4 binders through molecular docking and dynamics simulations using REFRAME and ZINC15 libraries. These agents altered chromatin structure and morphology in a manner comparable to BB-Cl-amidine, with amodiaquine and pyronaridine showing greater efficacy. This approach highlights the utility of computational-experimental integration for repurposing approved drugs for PAD4 inhibition, thereby reducing development timelines and clinical translation risks.

Notably, the effect of PAD4 inhibition on host antimicrobial defense remains a topic of ongoing investigation. Martinod et al. [334] reported no significant changes in bacteremia between *PAD4*-deficient and wild-type mice in the CLP model, alongside reduced inflammation and coagulation and improved survival in endotoxemia. Similarly, Wu et al. [335] observed no increase in bacterial burden in PAD4-inhibited mice in a *Pseudomonas aeruginosa* pneumonia model. However, a 2025 study employing a fecal-slurry peritonitis model of sepsis yielded inconsistent results [336]. Cani et al. [336] observed a trend toward higher bacterial loads in peritoneal lavage fluid and blood in *PAD4*-deficient septic mice. Furthermore, in female *PAD4*-deficient septic mice, very low survival rates were observed compared to their wild-type septic counterpart, and it was accompanied by more severe lung injury. The study also found that combined antibiotic treatment abolished the survival disadvantage associated with *PAD4* deficiency and sex differences. These findings suggest that while PAD4 inhibition offers therapeutic benefit, adjunctive antimicrobial therapy may be necessary to mitigate infection-related risks. Additionally, the timing of PAD4 inhibitor administration appears to be a critical factor influencing therapeutic outcomes. Holthaus et al. [337] found that early administration during acute myocardial infarction worsened cardiac damage, whereas delayed treatment improved remodeling and long-term survival. These results underscore the need for detailed evaluation of toxicity, timing, and dosing strategies. Continued development of PAD4 inhibitors with optimized profiles and direct comparative studies will be essential for identifying viable clinical candidates.

#### Pore-forming inhibitors

Cyclosporine A (CsA), an US Food and Drug Administration (FDA)-approved immunosuppressant, is widely used for preventing graft rejection and managing autoimmune conditions [338, 339]. As the gold-standard mPTP inhibitor, CsA disrupts the interaction between cyclophilin D and ATP synthase, thereby inhibiting mPTP formation, reducing mtDNA leakage, and mitigating inflammation [340, 341]. Multiple studies have demonstrated that CsA preserves mitochondrial function and decreases mtDNA levels, thereby reducing systemic inflammation, organ dysfunction, and mortality in sepsis models [342–345]. Recently, Xu et al. [346] reported that CsA also suppresses ROS-mediated NETs formation by inhibiting glucose-6-phosphate dehydrogenase, a key enzyme in the pentose phosphate pathway. These findings provide further insight into the mechanisms by which CsA limits cfDNA release. Nonetheless, long-term use of CsA has been associated with toxicities affecting the heart, liver, kidneys, and central nervous system [347]. Therefore, careful toxicity assessment and close monitoring of organ function are essential when considering CsA for sepsis treatment.

Disulfiram is an alcohol withdrawal drug that has been used clinically for decades [348]. In 2020, Hu et al. [349] first reported that disulfiram covalently modifies Cys191/Cys192 of GSDMD, inhibiting its pore formation and thereby blocking pyroptosis and cytokine release. In preclinical models of sepsis, disulfiram significantly decreased inflammatory cytokine levels and improved survival outcomes [349]. Subsequent work by Silva et al. [350] demonstrated that disulfiram effectively suppressed NET release by targeting GSDMD, thereby alleviating systemic inflammation and organ dysfunction associated with sepsis. Similar findings were reported in 2 independent studies on SARS-CoV-2 infection, where disulfiram inhibited GSDMD-dependent NETosis, exerting both anti-inflammatory and anticoagulant effects [351, 352]. More recently, Zhao et al. [353] uncovered a novel mechanism wherein disulfiram inhibits NETosis by blocking GSDMD-mediated MMO, thereby preventing mtDNA leakage and downstream cGAS-STING-dependent NET formation. To enhance its therapeutic potential, nanoparticle (NP)-based delivery systems have been developed to optimize disulfiram’s pharmacokinetic profile [354]. Beyond its NET-inhibitory properties, disulfiram exhibits anti-inflammatory and antimicrobial effects, further supporting its potential utility in sepsis. It suppresses NF-κB signaling through metal ion chelation and inhibits NLRP3 inflammasome activation by stabilizing lysosomal membranes, thus reducing pro-inflammatory cytokine production [355, 356]. Disulfiram also demonstrates antimicrobial activity against a broad range of pathogens, including bacteria [357], viruses [358], parasites [359], and Borrelia burgdorferi [360]. Despite its promising therapeutic potential in sepsis, the clinical application of disulfiram remains limited by several challenges. Notably, its pharmacokinetic profile is insufficiently defined, with considerable interindividual variability in metabolic and elimination rates, complicating the prediction of efficacy and safety in clinical settings [361]. Furthermore, disulfiram exhibits a complex safety profile, as high doses have been associated with serious adverse effects, including hepatotoxicity [362], neurotoxicity [363], and psychiatric disturbances [364]. Future studies should elucidate the pharmacokinetic mechanisms of disulfiram and rigorously evaluate its efficacy and safety for sepsis treatment at clinically relevant doses.

Other pore-forming inhibitors, including caspase [365], VDAC [366], and BAX/BAK [367] targeted agents, have similarly been shown to have pre-clinical potential to suppress cfDNA release and thereby alleviate sepsis-related inflammation and organ injury. The future work could focus on investigating the efficacy, safety, and combination strategies of various inhibitors to develop more targeted therapeutic candidates for the precision treatment of sepsis.

#### Antioxidants

Among conventional antioxidants, vitamin C has demonstrated particular advantages in sepsis intervention due to its potent ROS-scavenging capacity. It directly neutralizes ROS and promotes regeneration of endogenous antioxidants such as vitamin E, thereby supporting redox homeostasis [368]. Additionally, vitamin C inhibits ROS and RNS generation by suppressing NOX activation and reducing iNOS expression [369]. In experimental sepsis models, vitamin C significantly attenuated inflammatory responses, limited organ damage, and improved survival rates [370]. One of the key protective mechanisms may involve suppression of NETosis and consequent reduction in circulating cfDNA levels [371]. Interestingly, vitamin C exhibits a concentration-dependent effect on cfDNA: low doses (1 mmol/L) inhibit NET formation and decrease cfDNA release, whereas high doses (≥ 5 mmol/L) can induce extracellular H_2_O_2_ production, potentially enhancing cfDNA release [372, 373]. In clinical contexts, the “metabolic resuscitation protocol” combining vitamin C, hydrocortisone, and thiamine has shown promise in reducing sepsis-related organ dysfunction and lowering mortality from 40.4% to 8.5% in initial trials [374]. Furthermore, a post hoc analysis of the vitamin C infusion for treatment in sepsis-induced acute lung injury (CITRIS-ALI) trial found that patients receiving high-dose (50 mg/kg) intravenous vitamin C had significantly lower plasma cfDNA levels after 48 h, correlating with reduced 28-day mortality [375]. However, recent large-scale multicenter randomized controlled trials have failed to confirm these clinical benefits, leading to ongoing debate regarding vitamin C’s effectiveness in sepsis management [376–378]. Future investigations should focus on refining treatment protocols about dosage, timing, and duration to clarify its therapeutic role.

Mitochondria-targeted antioxidants, modified with lipophilic cations, offer enhanced efficacy by accumulating at primary ROS-generation sites within mitochondria [379, 380]. This mitochondrial specificity improves their antioxidant potency while minimizing off-target distribution [379, 380]. MitoQ, which links ubiquinone to a tetraphenylphosphonium cation, achieves mitochondrial concentrations several 100-fold greater than cytoplasmic levels [381]. Mechanistic studies reveal that MitoQ not only scavenges ROS efficiently but also activates protective signaling pathways. It induces NRF-2 pathway activation, thereby upregulating antioxidant genes such as *HMOX1*, *NQO1*, and *GCLM*, and mitigating LPS-induced oxidative stress and inflammation in intestinal tissues [382]. Additionally, MitoQ modulates cell death and autophagy pathways via the phosphoinositide 3-kinase (PI3K)/protein kinase B (Akt)/glycogen synthase kinase 3β (GSK-3β)-mammalian target of rapamycin axis, improving outcomes in sepsis-induced lung injury [383]. A recent study has shown that MitoQ preserves mitochondrial integrity, reduces mtDNA oxidation, and limits its release, contributing to reduced cfDNA burden [384]. In both in vitro and in vivo sepsis models, MitoQ suppressed oxidative stress and inflammation, maintained mitochondrial membrane potential and cell viability, and improved liver and kidney function [385]. Similar protective effects have been reported in sepsis-associated cardiac [386], pulmonary [383], intestinal [382], and skeletal muscle injury [387]. Other mitochondria-targeted antioxidants, such as MitoVitE, MitoTempol, and SS-31, have also shown potential for mitochondrial protection in sepsis, although their specific effects on cfDNA release remain to be fully established [388–390]. Notably, agents like MitoQ and MitoTempol demonstrate efficacy across a broad therapeutic window, addressing the clinical challenge of delayed diagnosis and treatment initiation in sepsis [387, 389]. Currently, MitoQ is undergoing phase II clinical trials as an oral therapy for hepatitis C [379], whereas dedicated clinical studies in sepsis remain an urgent priority.

In addition to synthetic antioxidants, certain natural compounds also exhibit mitochondria-directed activity. Melatonin, an endogenous antioxidant, exerts its protective effects through several mechanisms, including direct neutralization of free radicals, stimulation of antioxidant enzyme activity, chelation of transition metals, and suppression of free-radical production [391]. A notable feature of melatonin is its strong mitochondrial accumulation [392]. In septic rat models, its anti-inflammatory, antioxidant, and mitochondria-protective functions are on par with those of synthetic mitochondria-targeted agents such as MitoQ and MitoE [388]. Moreover, the mechanisms by which melatonin inhibits cfDNA release are comparatively well elucidated, and its therapeutic evaluation in sepsis is more advanced than that of synthetic counterparts. An experimental study indicates that melatonin decreases ROS production, thereby reducing chemokine expression, neutrophil infiltration, and NET formation [393]. It has further been reported to suppress NET release and inhibit AIM2 inflammasome activation, thereby alleviating SA-ALI [37]. A 2025 study demonstrated that melatonin attenuates SA-ALI by limiting cf-mtDNA release and blocking STING activation [222]. Beyond these effects, melatonin has been shown to protect multiple organs, including the heart [394], lungs [395], liver [396], kidneys [397], and brain [398] from sepsis-induced injury, while also lowering mortality in septic animal models. Currently, melatonin has entered phase II clinical trials for sepsis treatment. Clinical data show that melatonin significantly lowers markers of oxidative stress and inflammation in septic patients, resulting in shorter hospital stays, lower SOFA scores, and reduced mortality [399]. Comparable protective effects have also been reported in cases of neonatal sepsis [400]. Notably, melatonin has demonstrated a favorable safety profile across these clinical investigations [399, 400]. While early findings indicate potential therapeutic benefits, the available clinical evidence remains limited and is constrained by small sample sizes and variable study quality [401]. Moreover, a 2024 network meta-analysis identified possible publication bias in melatonin-related research, suggesting that its efficacy in sepsis may have been overestimated [402]. Therefore, future research should prioritize designing and conducting large-scale, high-quality randomized controlled trials to provide high-level evidence for melatonin’s clinical application. Another critical challenge for the clinical application of melatonin lies in optimizing its therapeutic regimen. Evidence regarding the appropriate dosing, route of administration, timing, and duration of treatment in sepsis remains preliminary, with no standardized protocols or strong clinical validation currently available [403–405]. Further studies are urgently required to establish dose–response relationships and elucidate pharmacokinetic characteristics, thereby enabling the development of evidence-based therapeutic guidelines for melatonin in sepsis.

### Promoting cfDNA clearance

Under physiological conditions, cfDNA is eliminated mainly through 3 routes: uptake by the hepatic and splenic reticuloendothelial systems, passive filtration by the kidneys, and enzymatic degradation mediated by plasma DNases [406]. In the setting of sepsis or severe infection, however, these clearance pathways become compromised, leading to pronounced accumulation of cfDNA in circulation. Clinical observations indicate that DNase I levels in septic patients remain consistently low for up to 28 d, accompanied by decreased DNase activity [407–409]. The reduction in DNase function and the consequent impairment of cfDNA/NETs clearance during sepsis are thought to result from splenocyte death and the release of actin, which acts as a natural inhibitor of DNase I [409]. The latest research suggests that impaired hepatic clearance, rather than excessive cellular death, may be the primary contributor to cfDNA accumulation during sepsis [60]. In addition, the plasma cfDNA levels in patients with SA-AKI and those requiring RRT are significantly higher, suggesting that impaired renal clearance function may be involved in the defect of cfDNA clearance [59]. Growing evidence indicates that impaired cfDNA clearance plays a significant role in the pathogenesis of cfDNA-associated diseases. For instance, accumulation of dsDNA resulting from DNase II deficiency has been identified as a key mechanism driving neuroinflammation and cognitive impairment [410, 411]. Moreover, multiple studies have shown that the pathogenesis of systemic lupus erythematosus is closely related to DNase I deficiency and impaired cfDNA clearance [412–414]. In the context of sepsis and severe infection, impaired clearance of cfDNA and NETs has been closely associated with disease progression and unfavorable clinical outcomes [409, 415]. Consequently, enhancing cfDNA clearance is considered a critical therapeutic strategy in cfDNA-targeted interventions for sepsis.

#### cfDNA scavengers

cfDNA scavengers neutralize circulating DNA through electrostatic adsorption and promote its metabolic clearance, thereby showing strong potential as anti-inflammatory and antithrombotic agents [416]. In contrast to conventional single-target molecular therapies, cfDNA scavengers act upstream by intercepting cfDNA-driven pathological processes at their origin [417]. Moreover, they selectively inhibit nucleic acid-induced inflammatory activation while sparing host responses to other pathogen-associated signals, thus preserving essential antimicrobial defenses [417, 418]. At present, cationic polymers and cationic NPs (cNPs) represent the most promising cfDNA scavengers under investigation for therapeutic use in sepsis.

Typical cationic polymers, such as polyethylenimine and polyamidoamine (PAMAM), use surface polyamine groups to generate positive charges, enabling electrostatic adsorption to the phosphate backbone of cfDNA, and effectively neutralizing its pro-inflammatory and pro-thrombotic activities [419, 420]. Cationic polymers mitigate cfDNA-driven inflammation through complementary extracellular and intracellular mechanisms. On the extracellular level, they electrostatically bind cfDNA, thereby preventing TLR9 activation and limiting cellular uptake of cfDNA [419, 421]. Intracellularly, by exploiting the proton sponge effect, they facilitate the release of cfDNA from TLR9-rich endosomes and redirect it into compartments that are inert to nucleic acid sensing, effectively dampening downstream signaling pathways [421]. In experimental models of trauma and hemorrhagic shock, hexadecylmethylammonium bromide markedly decreased circulating cfDNA levels, suppressed TLR9 pathway activation, and improved multi-organ function [422]. Similarly, PAMAM-G3 sequestered cfDNA to inhibit STING activation, resulting in reduced inflammatory responses in oral lichen planus [423]. Beyond inflammation, cationic polymers neutralized the negative charge of cfDNA, thereby blocking its role in activating coagulation pathways and driving thrombus formation [420]. Notably, PAMAM-G3 was able to inhibit thrombosis effectively without increasing bleeding risk [424]. While their anti-inflammatory and antithrombotic activities have been experimentally validated, the clinical translation of cationic polymers faces several challenges. For example, unshielded cationic surfaces readily disrupt cell membranes and induce blood cell injury [425, 426]. Although modifications to parameters such as charge density, molecular weight, and degree of branching can reduce cytotoxicity, these alterations may also decrease the cfDNA-binding capacity of cationic polymers [420, 427]. Furthermore, certain cationic polymers display immunogenic properties, aggravating systemic inflammation through the activation of PRRs such as TLR2, TLR4, and the NLRP3 inflammasome [428–430]. Additional limitations include low charge density per unit mass, limited adsorption specificity, inadequate biodistribution targeting, and rapid clearance by the mononuclear phagocyte system (MPS), all of which compromise their in vivo stability and cfDNA-binding efficiency [431]. While strategies such as physicochemical parameter tuning [419, 432], biocompatibility modifications [433, 434], and incorporation of multiple binding mechanisms [435] have partially alleviated these issues, standalone polymeric cfDNA scavengers remain inadequate for sepsis therapy. Consequently, the majority of cationic polymers being developed for sepsis therapy are administered in NP formulations rather than as unmodified polymers.

cNPs have emerged as a major research focus in cfDNA-clearance therapy as their nanoscale size allows them to overcome the limitations of conventional cationic polymers. First, the nanoscale dimensions of cNPs confer the ability to clear cfDNA with high efficiency, durability, and specificity. Mechanistically, their high surface-to-mass ratio increases charge density, thereby enhancing cfDNA binding and clearance [436, 437]. Furthermore, maintaining cNPs within the 10 − 200 nm size range minimizes glomerular filtration and MPS-mediated phagocytosis, significantly prolonging circulation half-life [438–440]. Their nanoscale size also facilitates passive accumulation and retention at inflammatory sites through the enhanced permeability and retention effect [441, 442]. For instance, SiO_2_-poly(N,N-dimethylaminoethyl methacrylate) (PDMA) composite NPs have demonstrated greater cfDNA-binding capacity, extended circulation time, improved biocompatibility, and superior targeting compared with free PDMA [437]. Similarly, in septic mouse models, polyethylenimine-grafted zeolitic imidazolate framework NPs exhibited reduced cytotoxicity, enhanced cfDNA clearance, and improved therapeutic efficacy relative to unmodified polymers [443]. Second, the large specific surface area of cNPs offers ample room for surface modification, enabling diverse functionalization strategies [444]. Polyethylene glycol (PEG) coatings can effectively reduce nonspecific protein adsorption and MPS clearance, prolonging their circulation time [445, 446]. PEGylated cNPs, however, are subject to the accelerated blood clearance effect, whereby their elimination rate increases significantly upon repeated administration [447]. In contrast, cell membrane-coated NPs (MNPs) preserve the membrane proteins and biointerface properties of their source cells, enabling them to resist nonspecific adsorption and evade immune recognition [448]. Consequently, MNPs achieve substantially prolonged circulation times without inducing the accelerated blood clearance phenomenon [449]. Beyond circulation benefits, membrane coatings also endow cNPs with additional biofunctionalities. For example, platelet-membrane-coated and macrophage-membrane-coated NPs have demonstrated both anti-inflammatory and antibacterial activities in sepsis models [450, 451]. Moreover, MNPs enable active targeting through specific membrane proteins on their surfaces [448]. Complementary strategies to reduce toxicity while maintaining cfDNA-binding capacity include grafting hydrophilic groups (e.g., PEG, carboxyl moieties) onto cNPs [452] or modifying primary amines into secondary or tertiary forms, such as through imidazole-acetic acid modification [453, 454]. Moreover, constructing degradable architectures cross-linked with disulfide bonds offers a promising means of minimizing the long-term toxicity of cNPs [455]. Additionally, novel modification strategies are being developed to further augment the cfDNA-scavenging capacity of cNPs. Similar to cationic polymers, cNPs can reduce their dependence on positive charge by incorporating functional groups that form salt bridges or coordinate with cfDNA, thereby enhancing binding efficiency and stability under complex physiological conditions [456, 457]. In addition, pH-responsive designs enable cNPs to maintain a low-charge state at physiological pH, minimizing nonspecific adsorption, while protonation of amine groups in the acidic endosomal environment markedly strengthens cfDNA binding [458]. This adaptive binding profile allows cNPs to selectively target inflammatory sites and intracellular cfDNA, ensuring effective clearance and suppression of inflammation [458]. Beyond cfDNA scavenging, cNPs also act as delivery platforms for other sepsis therapeutics, supporting multi-target synergistic interventions [459]. For instance, Li et al. [460] developed multifunctional NPs (TMPP) composed of tannic acid, polymyxin B, and Mn^2+^, which simultaneously neutralized multiple inflammatory mediators (LPS, cfDNA, and ROS) and exhibited strong antibacterial properties, thereby markedly improving therapeutic efficacy in sepsis. In summary, cNPs hold immense potential for sepsis treatment. Future research should focus on the development of integrated cfDNA monitoring and therapy platforms, as well as the optimization of cNPs’ long-term safety profiles and manufacturing processes.

#### DNases and DNase-like artificial enzymes

DNases function by cleaving the phosphodiester bonds of the DNA backbone, thereby facilitating the direct degradation and clearance of circulating cfDNA [406]. Unlike cfDNA scavengers, DNases are not subject to binding saturation and the subsequent risk of cfDNA re-release [431]. DNase I is the principal enzyme applied in therapeutic contexts, with its safety and efficacy well established. Recombinant human DNase I (Pulmozyme®) remains the sole FDA-approved drug for degrading cfDNA and NETs [461]. By contrast, DNase II is active only under acidic conditions (pH 4.5 − 5.0) [462], while DNase III has limited application due to its inability to process oxidatively damaged DNA [463]. Currently, DNase I is regarded as a promising therapeutic approach for the immunomodulatory management of diverse cfDNA/NET-related diseases [464–466]. In sepsis animal models, DNase I administration has significantly reduced cfDNA levels, thereby improving inflammatory markers, coagulation parameters, and organ functions (liver, kidney, intestine, etc.), ultimately leading to markedly increased survival rates [467–469]. DNase I and DNase1L3 have also been shown to exert antibacterial effects by degrading mcfDNA within biofilms [470, 471]. Importantly, the timing of DNase I administration following CLP has a critical impact on its therapeutic efficacy. Mai et al. [472] reported that although administration at 2, 4, or 6 h post-CLP all reduced cfDNA levels in septic mice, the treatment outcomes varied. Early intervention at 2 h increased IL-6 levels and aggravated lung and kidney injuries, whereas later administration at 4 or 6 h effectively suppressed inflammation, alleviated organ damage, and limited bacterial spread [472]. Consistently, Hu et al. [42] demonstrated that DNase I given 5 h post-CLP efficiently cleared mtDNA, lowered TNF-α levels, and improved sepsis-induced intestinal barrier dysfunction. Another study further revealed that plasma cfDNA levels peaked at 5 h after sepsis induction, reaching about 20-fold higher than control levels, and remained elevated beyond 6 h [473]. Together, these results indicate that administering DNase I near the cfDNA levels peak may maximize therapeutic efficacy, while excessively early treatment may intensify inflammation and tissue injury. In combination therapies, DNase I and antibiotics demonstrated synergistic effects in sepsis treatment, evidenced by further improvements in histopathological scores, bacterial burden, and survival [474]. In contrast, combined administration of DNase I with LMW heparin not only failed to achieve synergistic effects but also decreased the activity of both agents [468].

While free DNase I demonstrates therapeutic potential in sepsis models, its unfavorable pharmacokinetics present major barriers to clinical translation. Owing to its small molecular diameter (5.7 nm), it is rapidly cleared by renal filtration, resulting in a short plasma half-life of just 3 − 4 h [475]. Moreover, free DNase I lacks stability under physiological conditions, as its enzymatic function is readily impaired by proteolytic degradation and remains highly sensitive to fluctuations in pH and ionic strength [476–478]. Its relatively weak binding affinity to cfDNA further limits degradation efficiency [431]. Consequently, free DNase I requires repeated dosing and is unable to sustain consistent therapeutic effectiveness. To address these bottlenecks, NP-based delivery strategies have become a research priority for enhancing DNase I stability and activity [479]. PEGylated polydopamine-like nanospheres (DNase-I pMNSs) maintained enzymatic activity for up to 36 h in 10% foetal bovine serum, markedly outperforming free DNase I in stability [480]. In septic mouse models, DNase I-loaded pMNSs markedly enhanced cfDNA clearance, attenuated inflammation, preserved organ function, and improved survival outcomes [480]. Similarly, Lee et al. [481] developed long-acting DNase I NPs that increased enzymatic activity in sepsis models, achieving more effective cfDNA degradation and greater therapeutic benefit compared with free DNase I. Encapsulation of DNase I within methylated, erythrocyte-membrane-coated liposomes also enabled stable and sustained degradation of NETs and cfDNA during sepsis [482]. To further augment cfDNA scavenging, Huang et al. [483] designed self-propelled DNase I/human serum albumin nanomotors via glutaraldehyde-mediated conjugation. These nanomotors actively navigated inflammatory microenvironments and rapidly localized to cfDNA, displaying superior catalytic efficiency and therapeutic efficacy relative to free DNase I. In another approach, Filipczak et al. [484] functionalized DNase I nanomicelles with the anti-histone antibody 2C5, thereby enhancing targeting to NETs and significantly improving degradation efficiency.

Given the poor stability and high production cost of natural DNase I, transition-metal-based nanozymes have emerged as attractive alternatives [485]. These nanozymes mimic DNase activity owing to their distinct electronic structures and coordination catalytic properties. Mechanistically, the unsaturated d-/f-orbitals of transition-metal ions coordinate with DNA phosphate groups and act as Lewis acids, thereby hydrolyzing phosphodiester bonds and enabling efficient cfDNA degradation [485]. Experimental studies have confirmed that transition-metal nanozymes effectively degrade cfDNA while exerting both anti-inflammatory and antibacterial effects [486–488]. Compared with natural DNase I, transition-metal-nanozymes offer notable advantages in stability, production cost, and recyclability. Their high thermal and storage stability, combined with facile surface modification, further support large-scale production and clinical translation [489]. Reportedly, CeO_2_ NPs combined superior thermal resilience with surface-renewable redox cycles, achieving enhanced stability and reduced application costs [490]. Czescik et al. [491] developed a multifunctional catalytic interface by functionalizing gold NPs with Zn(II)-coordinated 1,4,7-triazacyclononane. This configuration facilitated activation of DNA phosphate groups through metal coordination, stabilized the reaction transition state via guanidinium-arginine interactions, and enabled nucleophilic attack by serine hydroxyl groups, significantly enhancing cfDNA-cleavage efficiency. Importantly, nanozymes can also be engineered with pathogen-detection functions to assess therapeutic responses. For instance, the aggregation-induced emission artificial enzyme designed by Han et al. [488], exhibited both DNase-mimetic activity and fluorescence-tracing capability. It could dynamically track microbiota changes while degrading cfDNA within bacterial biofilms. Despite their promising features, transition-metal-based nanozymes still encounter several limitations. First, their catalytic efficiency remains significantly lower than that of natural DNase I [485]. For example, the cleavage rate of FAM-A15 by an equivalent amount of CeO_2_ NPs has been reported to be only 1/100 of that achieved by the natural enzyme at 37 °C [490]. Second, nanozymes tend to aggregate or precipitate under physiological conditions or due to surface charge effects, which decreases both their stability and catalytic activity [492]. Furthermore, concerns regarding potential long-term accumulation and toxicity of metal components in vivo necessitate comprehensive safety assessments and the development of appropriate surface modifications [493]. Therefore, future research should focus on enhancing the catalytic performance of transition metal nanozymes and conducting rigorous in vivo safety studies to support their clinical translation.

## Conclusions

Sepsis is a critical clinical syndrome marked by systemic inflammation and coagulation disturbances, frequently resulting in life-threatening multi-organ dysfunction. Despite its status as a leading global cause of morbidity and mortality, progress in clinical management has remained limited. A primary obstacle in advancing sepsis care is the absence of precision medicine approaches, underscoring the urgent need for diagnostic and therapeutic strategies that directly target its underlying pathological mechanisms.

In recent years, numerous studies have thoroughly elaborated on the molecular mechanisms of cfDNA in sepsis pathology, particularly inflammation activation and coagulation disorders [22, 26, 27]. These studies have strengthened the feasibility and potential value of cfDNA as a molecular intervention target for sepsis. Notably, cfDNA does not always play a negative role in sepsis pathology. Research has shown that cfDNA may contribute positively to the host’s anti-infection defense by forming the reticular structure of NETs [78]. Meanwhile, the controversy regarding the roles of PAD4 inhibition in impairing the host’s anti-infection function partially confirms the potential benefits of cfDNA. In recent studies, *PAD4*-deficient female septic mice were observed to exhibit a decline in bacterial clearance ability, along with aggravated lung injury and increased mortality [336]. Although there is currently insufficient evidence to quantify the survival benefits of cfDNA in clearing pathogens through NETs, the findings suggest that cfDNA plays a positive role in sepsis pathology. Therefore, when developing cfDNA-targeted therapies in the future, the protective effects of cfDNA should be preserved as much as possible by optimizing administration timing, dosage, and treatment cycles.

In addition, the metabolic kinetics of cfDNA in sepsis have always been the core theoretical basis for the development of cfDNA-targeted sepsis therapies. The elucidation of the release mechanisms of cfDNA has given rise to numerous therapies, including PAD4 inhibitors, pore-forming inhibitors, and antioxidants. These therapies aim to block key molecular events in cfDNA generation, thereby reducing its extracellular release. The study of cfDNA clearance kinetics has laid the theoretical foundation for the application of DNases and cfDNA scavengers in sepsis treatment. Overall, the cfDNA clearance strategy appears more promising as the future development direction of cfDNA-targeted therapy in sepsis. Drugs targeting cfDNA generation may be more suitable as adjuvant medications to improve clinical outcomes. On the one hand, the sources and release mechanisms of cfDNA are highly complex. Single-target interventions are not only insufficient to completely block pathological cfDNA release but may also cause adverse reactions through interactions with other pathways. In contrast, cfDNA clearance therapy can directly and non-specifically eliminate cfDNA from all sources, thereby blocking the pathological effects it drives. On the other hand, as the phenomenon of impaired cfDNA clearance mechanisms becomes increasingly characterized, the cfDNA burden in sepsis patients is shifting from “excessive production” to “failed clearance”. Recent research has highlighted that the main reason for abnormal cfDNA accumulation during sepsis is not excessive cell death but impaired liver clearance [60]. Currently, aided by nanoscale effects and surface-modification strategies, nanotechnology is accelerating the development of cfDNA scavengers. These cfDNA scavengers not only display optimized pharmacokinetic properties and enhanced clearance efficiency but also hold promise as multi-target drug carriers and theranostic platforms.

Notably, in the field of sepsis monitoring, cfDNA has emerged as a pivotal biomarker for dynamic surveillance and precision therapeutics due to its unique biological origins, pathological characteristics, and epigenetic signatures. At present, the integration of high-throughput sequencing, tissue-of-origin profiling, and advanced bioinformatics is accelerating the clinical translation of cfDNA surveillance technologies. Looking forward, TGS, ML, and portable NAT technologies are anticipated to serve as key drivers in the development of personalized, intelligent, and multi-dimensional sepsis monitoring platforms. However, challenges such as limited model interpretability, data privacy concerns, and high implementation costs are expected to present significant obstacles to the widespread adoption of cfDNA-targeted surveillance strategies. In conclusion, cfDNA has emerged as a promising target in precision medicine for sepsis and holds substantial potential to transform the current clinical management paradigm of this life-threatening condition.

## Data Availability

Not applicable.

## Abbreviations

AIM2: Absent in melanoma 2
APACHE II: Acute physiology and chronic health evaluation II
ASC: Apoptosis-associated speck-like protein containing a caspase recruitment domain
ATP: Adenosine triphosphate
BAK: Bcl-2 antagonist or killer
BAX: Bcl-2-associated X protein
Bcl-2: B-cell lymphoma-2
BID: BH3-interacting domain death agonist
CARD: Caspase recruitment domain
cfDNA: Cell-free DNA
cf-mtDNA: Cell-free mitochondrial DNA
cGAMP: Cyclic GMP-AMP
cGAS: Cyclic GMP-AMP synthase
CLP: Cecal ligation and puncture
cNPs: Cationic nanoparticles
CpG: Cytidine-phosphate-guanosine
CRP: C-reactive protein
CsA: Cyclosporine A
Cyt c: Cytochrome c
DAMPs: Damage-associated molecular patterns
DIC: Disseminated intravascular coagulation
DNase: Deoxyribonuclease
DRP1: Dynamin-related protein 1
dsDNA: Double-stranded DNA
ER: Endoplasmic reticulum
eTLR9: Endosomal TLR9
FVIIa: Activated factor VII
FXI: Factor XI
FXII: Factor XII
FXIIa: Activated factor XII
GSDM: Gasdermin
HIN: Hematopoietic interferon-inducible nuclear proteins domain
HK: High-molecular-weight kininogen
HMW-cfDNA: High-molecular-weight cfDNA
ICU: Intensive care unit
IFN: Interferon
IL: Interleukin
IMM: Inner mitochondrial membrane
IRF: Interferon regulatory factor
LMW-cfDNA: Low-molecular-weight cfDNA
LPS: Lipopolysaccharide
MAVS: Mitochondrial antiviral signaling protein
mcfDNA: Microbial cell-free DNA
mcfDNA-seq: McfDNA sequencing
MFN: Mitofusin
ML: Machine learning
MMO: Mitochondrial membrane opening
MMP: Mitochondrial membrane permeabilization
MNPs: Cell membrane-coated nanoparticles
MODS: Multiple organ dysfunction syndrome
MOMP: Mitochondrial outer membrane permeabilization
MPS: Mononuclear phagocyte system
MPT: Mitochondrial permeability transition
mPTP: Mitochondrial permeability transition pore
MQC: Mitochondrial quality control
mtDNA: Mitochondrial DNA
mtRNS: Mitochondrial reactive nitrogen species
mtROS: Mitochondrial ROS
MyD88: Myeloid differentiation primary response 88
NAT: Nucleic acid testing
nDNA: Nuclear DNA
NE: Neutrophil elastase
NET-DNA: Neutrophil extracellular trap-derived DNA
NETs: Neutrophil extracellular traps
NF-κB: Nuclear factor kappa-B
mNGS: Metagenomic next-generation sequencing
NLRP3: NOD-like receptor protein 3
NOX: Nicotinamide adenine dinucleotide phosphate hydrogen oxidase
NRF: Nuclear respiratory factor
NTD: N-terminal domain
ODNs: Oligodeoxynucleotides
OMM: Outer mitochondrial membrane
OPA1: Optic atrophy 1
ox-mtDNA: Oxidised mitochondrial DNA
PAD: Peptidylarginine deiminase
PAMAM: Polyamidoamine
PAMPs: Pathogen-associated molecular patterns
PCR: Polymerase chain reaction
PCT: Procalcitonin
PEG: Polyethylene glycol
PGC-1α: Peroxisome proliferator-activated receptor γ coactivator-1α
PINK1: PTEN-induced putative kinase 1
PK: Prekallikrein
PRRs: Pattern recognition receptors
PYD: Pyrin domain
ROS: Reactive oxygen species
RRT: Renal replacement therapy
SA-AKI: Sepsis-associated acute kidney injury
SA-ALI: Sepsis-associated acute lung injury
SAE: Sepsis-associated encephalopathy
SIRS: Systemic inflammatory response syndrome
SOFA: Sequential Organ Failure Assessment
STING: Stimulator of interferon genes
sTLR9: Surface Toll-like receptor 9
TF: Tissue factor
TFAM: Mitochondrial transcription factor A
TFPI: Tissue factor pathway inhibitor
TGS: Third-generation sequencing
TLR: Toll-like receptor
TNF: Tumor necrosis factor
TRAF: Tumor necrosis factor receptor-associated factor
VDAC: Voltage-dependent anion channel
vWF: Von Willebrand factor
WBs: Wearable biosensors
